# The association between circulating 25-hydroxyvitamin D metabolites and type 2 diabetes in European populations: A meta-analysis and Mendelian randomisation analysis

**DOI:** 10.1371/journal.pmed.1003394

**Published:** 2020-10-16

**Authors:** Ju-Sheng Zheng, Jian’an Luan, Eleni Sofianopoulou, Stephen J. Sharp, Felix R. Day, Fumiaki Imamura, Thomas E. Gundersen, Luca A. Lotta, Ivonne Sluijs, Isobel D. Stewart, Rupal L. Shah, Yvonne T. van der Schouw, Eleanor Wheeler, Eva Ardanaz, Heiner Boeing, Miren Dorronsoro, Christina C. Dahm, Niki Dimou, Douae El-Fatouhi, Paul W. Franks, Guy Fagherazzi, Sara Grioni, José María Huerta, Alicia K. Heath, Louise Hansen, Mazda Jenab, Paula Jakszyn, Rudolf Kaaks, Tilman Kühn, Kay-Tee Khaw, Nasser Laouali, Giovanna Masala, Peter M. Nilsson, Kim Overvad, Anja Olsen, Salvatore Panico, J. Ramón Quirós, Olov Rolandsson, Miguel Rodríguez-Barranco, Carlotta Sacerdote, Annemieke M. W. Spijkerman, Tammy Y. N. Tong, Rosario Tumino, Konstantinos K. Tsilidis, John Danesh, Elio Riboli, Adam S. Butterworth, Claudia Langenberg, Nita G. Forouhi, Nicholas J. Wareham

**Affiliations:** 1 MRC Epidemiology Unit, University of Cambridge, Cambridge, United Kingdom; 2 Westlake Laboratory of Life Sciences and Biomedicine, Key Laboratory of Growth Regulation and Translational Research of Zhejiang Province, School of Life Sciences, Westlake University, Hangzhou, China; 3 MRC/BHF Cardiovascular Epidemiology Unit, Department of Public Health and Primary Care, University of Cambridge, Cambridge, United Kingdom; 4 National Institute for Health Research Blood and Transplant Research Unit in Donor Health and Genomics, Department of Public Health and Primary Care, University of Cambridge, Cambridge, United Kingdom; 5 VITAS, Oslo, Norway; 6 Julius Center for Health Sciences and Primary Care, University Medical Center Utrecht, Utrecht University, Utrecht, the Netherlands; 7 Navarra Public Health Institute, Pamplona, Spain; 8 Navarra Institute for Health Research (IdiSNA), Pamplona, Spain; 9 CIBER Epidemiology and Public Health (CIBERESP), Madrid, Spain; 10 Department of Epidemiology, German Institute of Human Nutrition Potsdam-Rehbruecke, Germany; 11 Public Health Division of Gipuzkoa, San Sebastian, Spain; 12 Department of Public Health, Aarhus University, Aarhus, Denmark; 13 International Agency for Research on Cancer, Lyon, France; 14 Center of Research in Epidemiology and Population Health, UMR 1018 Inserm, Institut Gustave Roussy, Paris South–Paris Saclay University, Villejuif, France; 15 Department of Clinical Sciences, Lund University, Malmö, Sweden; 16 Department of Population Health, Luxembourg Institute of Health, Strassen, Luxembourg; 17 Epidemiology and Prevention Unit, Milan, Italy; 18 Department of Epidemiology, Murcia Regional Health Council, Instituto Murciano de Investigación Biosanitaria Virgen de la Arrixaca, Murcia, Spain; 19 Department of Epidemiology and Biostatistics, School of Public Health, Imperial College London, London, United Kingdom; 20 Danish Cancer Society Research Center, Copenhagen, Denmark; 21 Unit of Nutrition and Cancer, Cancer Epidemiology Research Program, Catalan Institute of Oncology–Institut d’Investigació Biomédica de Bellvitge, L’Hospitalet de Llobregat, Barcelona, Spain; 22 Facultat Ciències Salut Blanquerna, Universitat Ramon Llull, Barcelona, Spain; 23 Division of Cancer Epidemiology, German Cancer Research Center (DKFZ), Heidelberg, Germany; 24 Department of Public Health and Primary Care, University of Cambridge, Cambridge, United Kingdom; 25 Cancer Risk Factors and Life-Style Epidemiology Unit, Institute for Cancer Research, Prevention and Clinical Network (ISPRO), Florence, Italy; 26 Department of Cardiology, Aalborg University Hospital, Aarhus, Denmark; 27 Dipartimento di Medicina Clinica e Chirurgia, University of Naples Federico II, Naples, Italy; 28 Public Health Directorate, Asturias, Spain; 29 Family Medicine Division, Department of Public Health and Clinical Medicine, Umeå University, Umeå, Sweden; 30 Andalusian School of Public Health (EASP), Granada, Spain; 31 Instituto de Investigación Biosanitaria de Granada, Universidad de Granada, Granada, Spain; 32 Unit of Cancer Epidemiology, Città della Salute e della Scienza di Torino University Hospital–University of Turin and Center for Cancer Prevention (CPO), Torino, Italy; 33 National Institute for Public Health and the Environment (RIVM), Bilthoven, the Netherlands; 34 Cancer Epidemiology Unit, Nuffield Department of Population Health, University of Oxford, Oxford, United Kingdom; 35 Azienda Sanitaria Provinciale, Ragusa, Italy; 36 Department of Hygiene and Epidemiology, University of Ioannina School of Medicine, Ioannina, Greece; 37 British Heart Foundation Cambridge Centre of Excellence, Division of Cardiovascular Medicine, Addenbrooke’s Hospital, Cambridge, United Kingdom; 38 Department of Human Genetics, Wellcome Trust Sanger Institute, Wellcome Trust Genome Campus, Hinxton, United Kingdom; Chinese University of Hong Kong, CHINA

## Abstract

**Background:**

Prior research suggested a differential association of 25-hydroxyvitamin D (25(OH)D) metabolites with type 2 diabetes (T2D), with total 25(OH)D and 25(OH)D_3_ inversely associated with T2D, but the epimeric form (C3-epi-25(OH)D_3_) positively associated with T2D. Whether or not these observational associations are causal remains uncertain. We aimed to examine the potential causality of these associations using Mendelian randomisation (MR) analysis.

**Methods and findings:**

We performed a meta-analysis of genome-wide association studies for total 25(OH)D (*N =* 120,618), 25(OH)D_3_ (*N =* 40,562), and C3-epi-25(OH)D_3_ (*N =* 40,562) in participants of European descent (European Prospective Investigation into Cancer and Nutrition [EPIC]–InterAct study, EPIC-Norfolk study, EPIC-CVD study, Ely study, and the SUNLIGHT consortium). We identified genetic variants for MR analysis to investigate the causal association of the 25(OH)D metabolites with T2D (including 80,983 T2D cases and 842,909 non-cases). We also estimated the observational association of 25(OH)D metabolites with T2D by performing random effects meta-analysis of results from previous studies and results from the EPIC-InterAct study. We identified 10 genetic loci associated with total 25(OH)D, 7 loci associated with 25(OH)D_3_ and 3 loci associated with C3-epi-25(OH)D_3_. Based on the meta-analysis of observational studies, each 1–standard deviation (SD) higher level of 25(OH)D was associated with a 20% lower risk of T2D (relative risk [RR]: 0.80; 95% CI 0.77, 0.84; *p <* 0.001), but a genetically predicted 1-SD increase in 25(OH)D was not significantly associated with T2D (odds ratio [OR]: 0.96; 95% CI 0.89, 1.03; *p =* 0.23); this result was consistent across sensitivity analyses. In EPIC-InterAct, 25(OH)D_3_ (per 1-SD) was associated with a lower risk of T2D (RR: 0.81; 95% CI 0.77, 0.86; *p <* 0.001), while C3-epi-25(OH)D_3_ (above versus below lower limit of quantification) was positively associated with T2D (RR: 1.12; 95% CI 1.03, 1.22; *p =* 0.006), but neither 25(OH)D_3_ (OR: 0.97; 95% CI 0.93, 1.01; *p =* 0.14) nor C3-epi-25(OH)D_3_ (OR: 0.98; 95% CI 0.93, 1.04; *p =* 0.53) was causally associated with T2D risk in the MR analysis. Main limitations include the lack of a non-linear MR analysis and of the generalisability of the current findings from European populations to other populations of different ethnicities.

**Conclusions:**

Our study found discordant associations of biochemically measured and genetically predicted differences in blood 25(OH)D with T2D risk. The findings based on MR analysis in a large sample of European ancestry do not support a causal association of total 25(OH)D or 25(OH)D metabolites with T2D and argue against the use of vitamin D supplementation for the prevention of T2D.

## Abstract 2

Using both observational and genetic analyses, Ju-Sheng Zheng and colleagues investigate the relationship between vitamin D metabolites and type 2 diabetes among European individuals.

## Author summary

**Why was this study done?:**

- There is ongoing uncertainty on whether the body’s vitamin D status indicated by blood 25-hydroxyvitamin D (25(OH)D) is relevant to the prevention of type 2 diabetes. There are conflicting findings from observational studies and a limited number of randomised controlled trials.
- Prior research did not distinguish between different metabolites of total 25(OH)D, including 25(OH)D_3_ and C3-epi-25(OH)D_3_, an isomer of 25(OH)D_3_.
- It is not clear whether the associations of 25(OH)D metabolites with type 2 diabetes are causal, with conflicting findings from prior Mendelian randomisation studies of total 25(OH)D and no previous Mendelian randomisation studies appraising 25(OH)D metabolites.

**What did the researchers do and find?:**

- The current research compared observational estimates of the association between 25(OH)D metabolites and incident type 2 diabetes with Mendelian randomisation estimates based on genetic instruments.
- Using multiple data sources, we performed genome-wide association studies among 120,618 individuals for total 25(OH)D, and among 40,562 individuals for the other vitamin D metabolites. Among participants of European descent, 10 genetic loci were identified for total 25(OH)D, 7 loci for 25(OH)D_3_ and 3 loci for C3-epi-25(OH)D_3_.
- In meta-analysis of observational studies, we found that each 1–standard deviation higher level of total 25(OH)D was associated with 20% lower risk of type 2 diabetes. The result was similar for 25(OH)D_3_, but for C3-epi-25(OH)D_3_, a positive association with type 2 diabetes was found.
- With up to 80,983 type 2 diabetes cases and 842,909 controls, we assessed the association of genetically predicted differences in total 25(OH)D and its metabolites with type 2 diabetes. Neither genetically predicted higher total 25(OH)D level nor genetically predicted higher levels of 25(OH)D metabolites were significantly associated with type 2 diabetes.

**What do these findings mean?:**

- There were conflicting findings for a link with type 2 diabetes for the observational analysis of biochemically measured 25(OH)D metabolites versus the genetically predicted levels of these metabolites.
- The null findings based on Mendelian randomisation analysis indicate that blood levels of 25(OH)D or its metabolites are not likely to be causal factors for the development of type 2 diabetes.
- The current findings together with other evidence from randomised controlled trials do not support the use of vitamin D supplementation for the prevention of type 2 diabetes.

## Introduction

Adequate vitamin D status is crucial for maintaining bone homeostasis, and interest in its potential beneficial roles in cardiometabolic diseases, including type 2 diabetes (T2D), has increased recently. Prospective epidemiological studies have consistently reported an inverse association between circulating 25-hydroxyvitamin D (25(OH)D, a blood marker of vitamin D status) and T2D risk [1,2], but whether there is a causal relationship between vitamin D status and T2D remains uncertain. Older randomised controlled trials (RCTs) reported no beneficial effect of vitamin D supplementation on T2D risk [3,4], but they were limited by issues of sub-group or post hoc analyses, inadequate dose, or inability to separate the effect of vitamin D and calcium. The results of the VITAL study for the secondary endpoint of T2D are still awaited, with the primary endpoints being cancer and cardiovascular disease [5]. With T2D as a primary endpoint, the D2d RCT recently reported that vitamin D_3_ supplementation at a dose of 4,000 IU per day among people at high risk for T2D did not result in a lower risk of T2D than placebo [6]. This evidence argues against a benefit of vitamin D_3_ supplementation for the prevention of T2D, but there are still unresolved issues.

The D2d trial included participants with confirmed prediabetes, so it remains unclear whether vitamin D supplementation may be more effective at earlier stages in the natural history of T2D, before beta cell dysfunction. However, designing a trial to examine the effects of early supplementation poses several challenges including long follow-up, adherence, and cost. The mean baseline 25(OH)D level was 70 nmol/l in D2d, with the majority of participants vitamin D replete, and the study was not adequately powered to examine potential effects of supplementation among participants with lower levels of 25(OH)D. Moreover, studies of supplementation do not directly assess the causal relationship of long-term body vitamin D status as assessed by blood 25(OH)D metabolite levels, which is relevant biologically given that blood levels reflect both dietary source and endogenous synthesis. Genetic Mendelian randomisation (MR) analysis is a complementary approach to RCTs that, subject to several assumptions, can enable the estimation of causal associations using data from observational studies [7,8]. Given the lack of definitive evidence from RCTs and the challenges of implementing an ideal RCT, MR studies can be a useful supplementary tool.

Previous MR analyses of 25(OH)D and T2D risk reported conflicting results, with some MR studies suggesting no association [1,9,10] but others reporting inverse associations [11,12]. A prior constraint was the use of a genetic instrument that included a limited set of up to 4 single nucleotide polymorphisms (SNPs) in genes involved in vitamin D synthesis and metabolism. Specifically, synthesis pathways involve *DHCR7* in 25(OH)D synthesis in the skin and *CYP2R1* in hepatic 25-hydroxylation, while metabolism pathways involve *GC* (or *DBP*; encoding vitamin D binding protein) in 25(OH)D transport and *CYP24A1* in 25(OH)D catabolism [13]. The inclusion of a greater number of genetic variants could generate a more powerful genetic instrumental variable for MR analysis. A further limitation to date has been the lack of a genetic instrument that can distinguish the 25(OH)D metabolites, including 25(OH)D_3_ and C3-epi-25(OH)D_3_, an isomer of 25(OH)D_3_ [14] that was positively associated with incident T2D [2]. There is no prior genome-wide association study (GWAS) of 25(OH)D_3_ to our knowledge, as past studies were restricted to analysing total 25(OH)D. Similarly, no GWAS to our knowledge has been performed for C3-epi-25(OH)D_3_.

Therefore, our objective was to assess the evidence for whether the association between total 25(OH)D and T2D is causal, using MR based on a genetic instrument derived from an updated meta-analysis of GWASs for total 25(OH)D. In addition, taking a similar approach, we also assessed the evidence to support any potentially causal associations with T2D for the vitamin D metabolites 25(OH)D_3_ and C3-epi-25(OH)D_3_ using a novel meta-analysis of GWASs for 25(OH)D_3_ and C3-epi-25(OH)D_3_.

## Methods

### Study design and populations

We adopted a multi-stage approach comprising 3 parts (defined in a prospective analysis plan; S1 Text): stage 1, GWASs of total 25(OH)D and vitamin D metabolites (25(OH)D_3_ and C3-epi-25(OH)D_3_); stage 2, MR analysis of T2D risk using GWAS-identified lead genetic variants; and stage 3, comparison of MR estimates with observational estimates on the association between vitamin D metabolites and T2D incidence.

**Fig 1:**
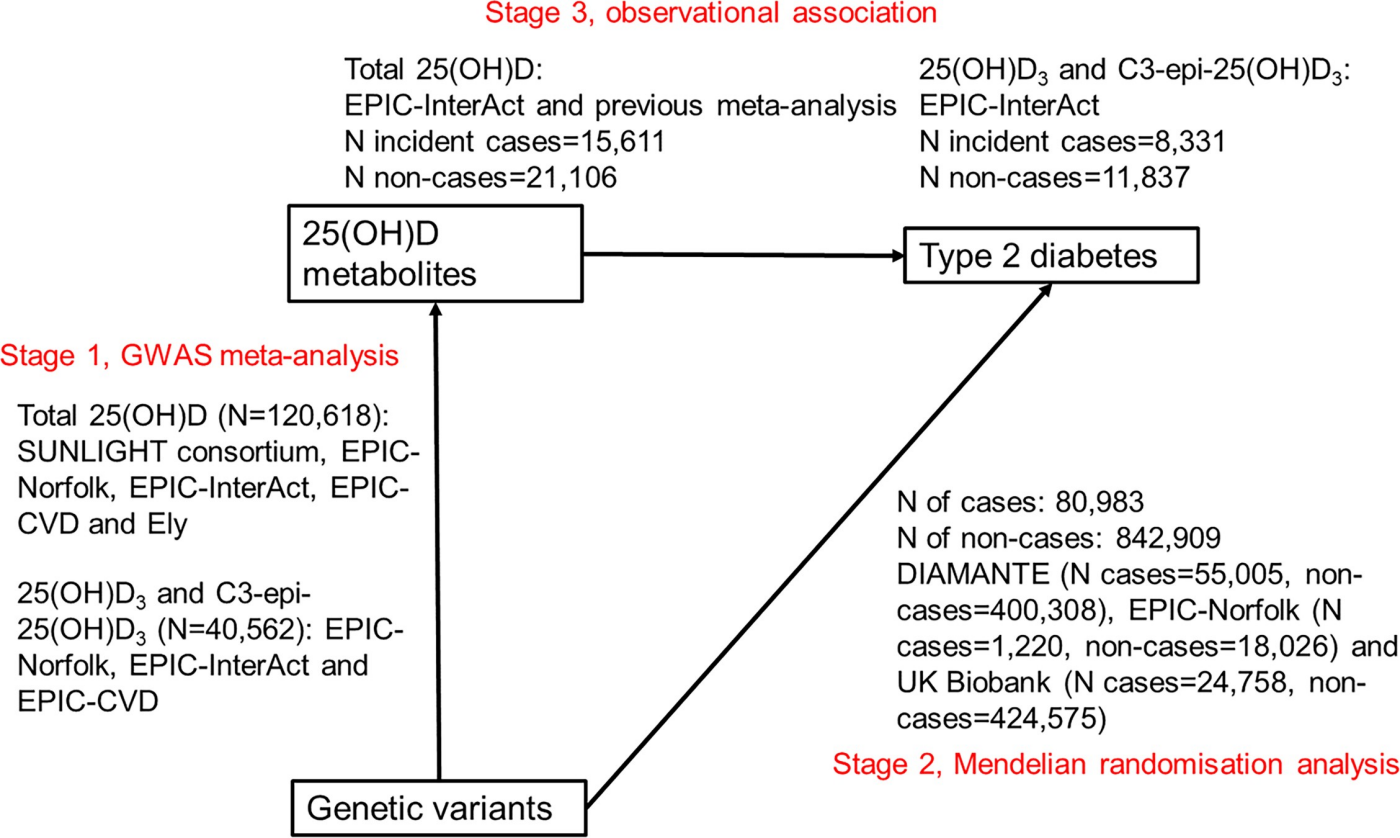
Design of the Mendelian randomisation study to estimate the causal association of 25-hydroxyvitamin D metabolites with type 2 diabetes. 25(OH)D, 25-hydroxyvitamin D; GWAS, genome-wide association study.

At the GWAS stage, for total 25(OH)D, we performed a meta-analysis of GWASs including 120,618 participants of European origin from a number of cohorts as shown in Fig 1: the European Prospective Investigation into Cancer and Nutrition (EPIC)–InterAct study (*n =* 18,078) [15], EPIC-Norfolk study (*n =* 10,231) [16], EPIC-CVD study (*n =* 12,253) [17], Ely study (*n =* 690) [18], and publicly available GWAS summary statistics from the SUNLIGHT consortium (*n =* 79,366) [19]. We excluded participants duplicated among the EPIC-InterAct study, EPIC-Norfolk study, and EPIC-CVD study. We then used the UK Biobank dataset (*n =* 410,826) to replicate the findings from the above GWAS meta-analysis of total 25(OH)D. For meta-analysis of GWASs of both 25(OH)D_3_ and C3-epi-25(OH)D_3_, we included up to 40,562 participants of European origin from the EPIC-InterAct study (*n =* 18,078), EPIC-Norfolk study (*n =* 10,231), and EPIC-CVD study (*n =* 12,253).

At the MR stage, we estimated the association of GWAS-identified lead genetic variants with T2D risk, performing a meta-analysis including participants (80,983 T2D cases, and 842,909 non-cases) from the DIAMANTE study (55,005 T2D cases, 400,308 non-cases) [20], UK Biobank (24,758 T2D cases, 424,575 non-cases) [21], and EPIC-Norfolk study (additional cases not included in DIAMANTE study: 1,220 T2D cases, 18,026 non-cases) [16].

At stage 3, as a comparison to MR results, we estimated the observational association of total 25(OH)D, 25(OH)D_3_, and C3-epi-25(OH)D_3_ with T2D in the EPIC-InterAct study (8,331 T2D cases, 11,837 non-cases), adapted from the analysis in our prior publication [2]. For total 25(OH)D, we combined the results from the EPIC-InterAct study with those from a previous meta-analysis [1].

### Ethics statement

Detailed description of each study is provided in S2 Text. All the studies included in the present analyses were approved by local ethical committees, and participants provided written informed consent. Specifically, EPIC-InterAct and EPIC-CVD were approved by local ethics committees in the participating countries and the institutional review board of the International Agency for Research on Cancer. EPIC-Norfolk was approved by the Norwich local ethics committee, and the Ely study was approved by the Ely local research ethics committee.

### Circulating 25(OH)D measurement and genome-wide genotyping

In the EPIC-InterAct study, EPIC-Norfolk study, and EPIC-CVD study, plasma 25(OH)D metabolites were measured using liquid chromatography–tandem mass spectrometry (LC-MS/MS) at VITAS (Oslo, Norway; a reference lab in Europe with a Vitamin D External Quality Assessment Scheme certificate) [2]. In the Ely study, serum total 25(OH)D concentrations were measured by radioimmunoassay. The GWAS summary statistics from the SUNLIGHT consortium included data from 31 studies, within which radioimmunoassay, LC-MS/MS, enzyme-linked immunosorbent assay (ELISA), or chemiluminescence immunoassay was used for the measurement of the 25(OH)D metabolites [19]. In the UK Biobank, serum total 25(OH)D was measured using chemiluminescence immunoassay. Methods of genome-wide genotyping in each study are presented in S2 Text.

### Statistical analysis

**GWASs.** For total 25(OH)D, we performed meta-analysis of 9 sets of GWASs (Table 1) including 4 in the EPIC-InterAct study (subcohort GWAS array [*n =* 3,844], subcohort core-exome array [*n =* 6,932], non-subcohort GWAS array [*n =* 3,188], and non-subcohort core-exome array [*n =* 4,114]), 1 in the EPIC-Norfolk study (*n =* 10,231), 2 in the EPIC-CVD study (subcohort [*n =* 887] and non-subcohort [*n =* 11,366]), 1 in the Ely study (*n =* 690), and 1 from the SUNLIGHT consortium (*n =* 79,366). For 25(OH)D_3_ and C3-epi-25(OH)D_3_ (as a binary variable: above versus below the lower limit of quantification [LLQ; 1 nmol/l]), the same datasets as total 25(OH)D were used except for the Ely study, where only total 25(OH)D was available. C3-epi-25(OH)D_3_ was treated as a binary variable as around half of values were below the LLQ (i.e., missing) in each individual cohort.

**Table 1:** Characteristics of the cohorts included in the genome-wide meta-analysis.

Variable	EPIC-InterAct subcohort GWAS array	EPIC-InterAct subcohort core-exome array	EPIC-InterAct non-subcohort GWAS array	EPIC-InterAct non-subcohort core-exome array	EPIC-Norfolk	EPIC-CVD subcohort	EPIC-CVD non-subcohort	Ely study
Participants included in the GWAS, *N*	3,844	6,932	3,188	4,114	10,231	887	11,366	690
Age (years), mean (SD)	50.6 (9.3)	52.2 (8.9)	54.4 (8.06)	55.7 (6.98)	61.4 (8.9)	53.4 (12.3)	58.6 (8.2)	53.4 (7.7)
BMI, kg/m^2^, mean (SD)	26.3 (4.4)	26.0 (4.1)	30.1 (4.7)	29.6 (4.77)	25.9 (3.6)	28.4 (4.8)	26.9 (4.2)	25.7 (3.97)
Female sex, *n* (%)	2,463 (64.1)	4,226 (61)	1,643 (51.5)	1,970 (47.9)	5,892 (57.6)	527 (59)	4,831 (43)	393 (57)
Plasma 25(OH)D (nmol/l), mean (SD)	42.02 (18.23)	42.43 (18.15)	38.68 (17.12)	37.95 (17.2)	57.33 (22.84)	38.01 (15.80)	41.27 (17.64)	58.58 (23.9)
Plasma 25(OH)D_3_ (nmol/l), mean (SD)	40.88 (17.43)	41.08 (17.38)	37.59 (16.26)	36.66 (16.42)	56.96 (22.84)	38.00 (15.83)	41.29 (17.82)	NA
Plasma C3-epi-25(OH)D_3_ (nmol/l), mean (SD)	2.13 (1.38)	2.17 (1.35)	2.18 (1.45)	2.19 (1.45)	2.31 (1.34)	1.98 (1.03)	2.13 (1.29)	NA
Plasma C3-epi-25(OH)D_3_ (binary, yes), percent	40.2	41.2	39.0	37.6	59.1	38.2	41.0	NA
Ratio of C3-epi-25(OH)D_3_ to 25(OH)D_3_, percent	4.38 (2.04)	4.43 (2.09)	4.77 (2.28)	4.85 (2.45)	4.01 (2.63)	4.20 (1.07)	4.41 (2.15)	NA
Genotyping chip	Illumina 660W-Quad BeadChip	Illumina HumanCoreExome array	Illumina 660W-Quad BeadChip	Illumina HumanCoreExome array	Affymetrix UK Biobank Axiom Array	Illumina HumanCoreExome array	Illumina HumanCoreExome array	Illumina HumanCoreExome array
Imputation panel	HRC	HRC	HRC	HRC	HRC	HRC	HRC	HRC
Number of GWAS SNPs*	7,737,656	7,693,434	7,739,029	7,686,676	7,716,054	7,705,977	7,692,630	8,171,690

*Number of GWAS SNPs indicates number of SNPs with minor allele frequency ≥ 1% within each cohort, imputation quality (info score) ≥ 4, and *p*-value for Hardy–Weinberg equilibrium ≥ 10^−6^.

25(OH)D, 25-hydroxyvitamin D; BMI, body mass index; GWAS, genome-wide association study; HRC, Haplotype Reference Consortium; SD, standard deviation; SNP, single nucleotide polymorphism.

For each participating cohort in the above GWAS discovery, standardised residuals of natural-log transformed 25(OH)D metabolites were calculated, adjusting for age, sex, BMI, season of blood collection, and study centre (where appropriate). Then, the GWAS was performed using linear regression with SNPTEST (v2.5.4) assuming an additive effect, adjusting for the first 10 genetic principal components of ancestry within each cohort. For the binary C3-epi-25(OH)D_3_ variable, the GWAS was performed using logistic regression (above versus below LLQ) with QUICKTEST (v6.5.2) assuming an additive effect, adjusting for age, sex, BMI, season of blood collection, study centre (where appropriate), and the first 10 genetic principal components of ancestry within each cohort.

For each of the 25(OH)D measures (total 25(OH)D, 25(OH)D_3_, and C3-epi-25(OH)D_3_), we performed fixed-effect inverse-variance-weighted (IVW) meta-analysis to combine our results with published GWAS summary statistics (from the SUNLIGHT consortium, only for total 25(OH)D) [19] using the software METAL [22]. The quality control thresholds were as follows: minor allele frequency ≥ 0.01, imputation info score ≥ 0.4, and *p*-value for Hardy–Weinberg equilibrium ≥ 1.0 × 10^−6^. Associated loci were identified using the conventional threshold for genome-wide statistical significance (*p* < 5 × 10^−8^). At each locus, the lead SNP was identified as the SNP with the lowest *p-*value within a 1 million base-pair window. To visualise the findings, we generated Manhattan plots and quantile–quantile plots for the genetic association using R package EasyStrata version 8.5, and regional association plots using LocusZoom software [23]. We used HaploReg v4.1 to explore annotations of the identified lead SNPs, including the nearest genes, eQTL, GRASP QTL, and previous GWAS hits [24].

We used UK Biobank to replicate our GWAS findings for total 25(OH)D. In the UK Biobank study, standardised residuals of natural-log transformed total 25(OH)D were calculated, adjusting for age, sex, BMI, season of blood collection, genotype chip, and aliquot number. GWAS analysis of total 25(OH)D was performed using a linear mixed model with BOLT-LMM, adjusting for the first 10 genetic principal components of ancestry.

#### Estimation of genetic correlations and variance explained

Genetic correlation of 25(OH)D variables with T2D and related glycaemic traits (fasting glucose, insulin, 2-hour glucose, homeostatic model assessment of insulin resistance [HOMA-IR], homeostatic model assessment of beta cell function [HOMA-B], and glycated haemoglobin [HbA1c]) was estimated with linkage disequilibrium score regression analysis using the meta-analysed GWAS summary statistics and publicly available datasets in the LD Hub platform [25,26]. Variance in 25(OH)D metabolites explained by the identified lead SNPs was estimated using linear regression models with individual-level data from the EPIC-Norfolk study and UK Biobank study. We also calculated the F-statistic in the EPIC-Norfolk study to evaluate the strength of the genetic instrument.

#### Observational analysis

For comparison to MR, we estimated the observational association between total 25(OH)D and T2D incidence by meta-analysing the results from a previous meta-analysis [1] together with results from the EPIC-InterAct study we previously published [2] (cases *n =* 15,611; non-cases *n =* 21,106). We also used the published effect estimate of T2D for 25(OH)D_3_ in the EPIC-InterAct study [2]. For the observational association between C3-epi-25(OH)D_3_ and T2D, we used results based on the EPIC-InterAct study, adapted from our prior analysis [2], to estimate the relative risk (RR) of T2D comparing those above versus below the LLQ. To minimise the possibility of residual confounding in the observational association between 25(OH)D and T2D by adiposity, we performed exploratory analyses examining the influence of additional adiposity-related covariates. Specifically, in the EPIC-InterAct study, we included a genetic risk score (GRS) for BMI (generated from 97 BMI-related genetic variants by summing up the number of risk alleles) [27], and quadratic terms for BMI and waist-to-hip ratio, to account for a potential non-linear association between adiposity and T2D.

#### MR analyses

We performed MR analysis to combine estimates of ‘SNP to 25(OH)D level’ and ‘SNP to T2D’ associations, to estimate the genetically predicted association of the 25(OH)D variable (either total 25(OH)D or 25(OH)D_3_) with T2D (odds ratio [OR] per 1-SD increase). Similarly, we estimated the association of genetically predicted higher C3-epi-25(OH)D_3_ level (above versus below the LLQ) with T2D risk. For each of the 25(OH)D variables, we used an IVW method, MR-Egger method, and weighted median method to pool the estimates from multiple SNPs [28,29]. We used MR-Egger regression to detect and adjust for potential unbalanced pleiotropy in the MR analysis, and we used the weighted median MR method to examine the robustness of the results and highlight the results if significant heterogeneity of the associations among different genetic variants was observed. We used the effect estimate from the 25(OH)D GWAS discovery cohort, not UK Biobank, for the above MR analyses, as there were a large number of overlapping UK Biobank samples in the T2D GWASs. In response to peer reviewer comments, we performed analyses using several other MR methods to test the robustness of our results (S1 Text), including MR-PRESSO [30], MR-RAPS [31], and MRMix [32], and we further performed a multivariable MR analysis [7] that jointly estimated the causal association of the highly related traits. In addition, we calculated the statistical power of the MR analysis, which suggested that our MR had 90% power to detect a 6% difference in T2D risk per 1-SD change in 25(OH)D (α = 0.05, assuming that the genetic instrument explains 3.95% of the variance of the exposure).

MR assumptions are violated if there is horizontal pleiotropy, i.e., MR is valid when the genetic instrument is associated only with the exposure, not other variables that may be potential confounders. To assess the plausibility of this assumption, we examined the association of the GWAS-identified lead SNPs with metabolic markers and lifestyle and demographic factors, using the PhenoScanner tool (v2) [33]. We performed secondary analyses stratifying the genetic variants into several groups according to their biological roles: for total 25(OH)D and 25(OH)D_3_, lead SNPs were divided into those in the vitamin D synthesis pathway (*CYP2R1* and *NADSYN1/DHCR7*), in the vitamin D metabolism pathway (*GC*, *CYP24A1*), and the others (*AMDHD1*, *SEC23A*, *PADI1*, *CRCT1*, *UGT1A5*, *SULT2A1*); for C3-epi-25(OH)D_3_, lead SNPs were divided into 2 groups: the *SDR9C7* group and the others (which overlapped with 25(OH)D genes).

We also performed MR analysis to examine the genetically predicted association of the 25(OH)D metabolites with glycaemic traits, including HOMA-IR, HOMA-B, HbA1c, fasting insulin, fasting glucose, and 2-hour glucose. R version 3.4.3 and Stata version 14.2 (StataCorp) were used for the above statistical analyses.

This study is reported as per the Strengthening the Reporting of Observational Studies in Epidemiology (STROBE) guideline (S1 STROBE Checklist).

## Results

### Total 25(OH)D and T2D

**Fig 2:**
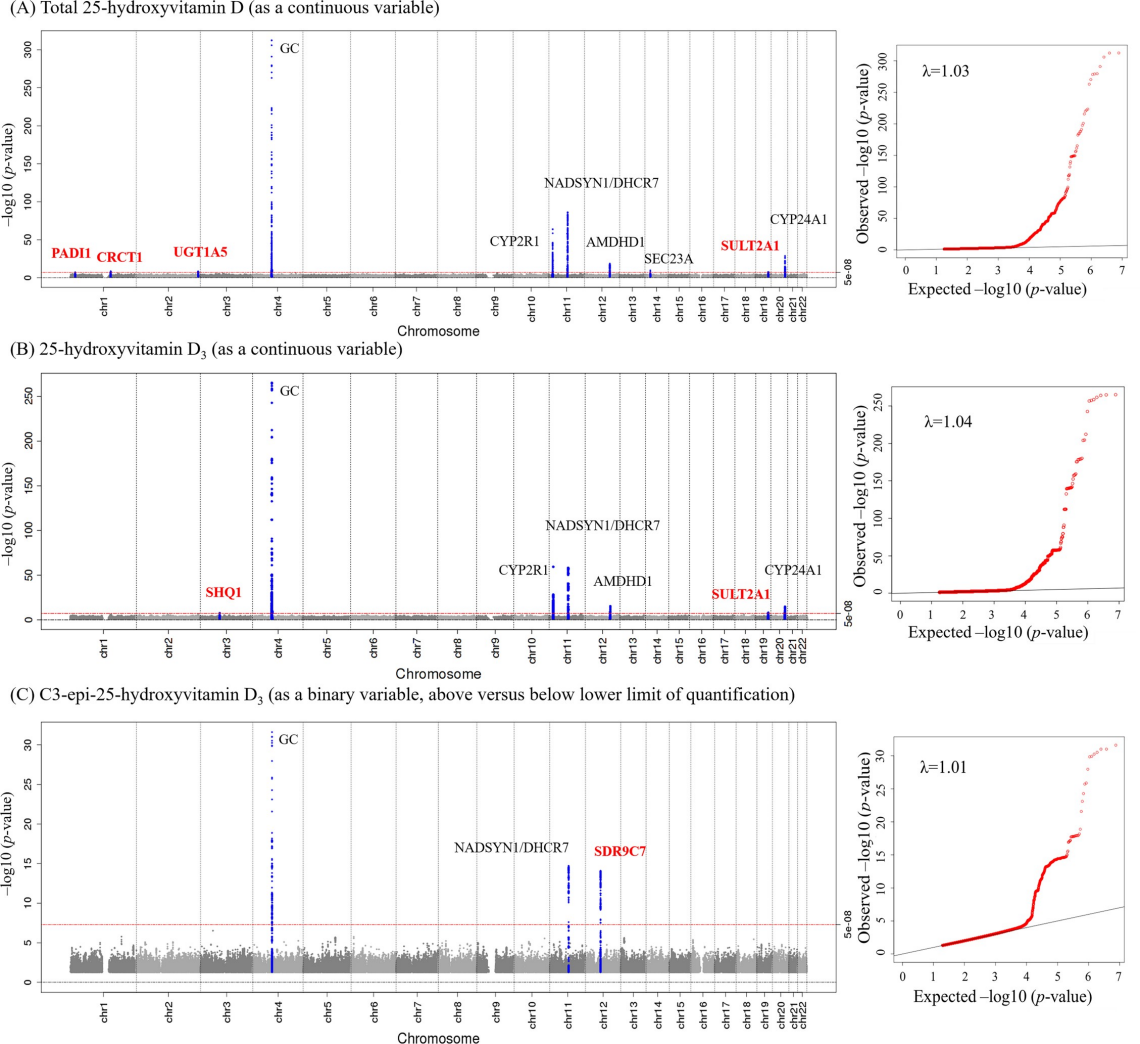
Genome-wide association of circulating 25-hydroxyvitamin D metabolites: Manhattan plot and quantile–quantile plot of all genetic variants from the meta-analysis. Manhattan plot and quantile–quantile plot for each of total 25-hydroxyvitamin D (A), 25-hydroxyvitamin D_3_ (B), and C3-epi-25-hydroxyvitamin D_3_ (C). Manhattan plot (left): SNPs are plotted on the *x*-axis according to their positions on each chromosome. The red line indicates the threshold for genome-wide significance (*p <* 5 × 10^−8^). Blue points represent SNPs in the ±100-kb region of a top hit. Loci are annotated with the gene names closest to the SNPs with lowest *p*-values (red indicates novel loci identified in the present study). Quantile–quantile plot (right): The *x*-axis shows the expected −log_10_ *p-*values, and the *y*-axis shows the observed −log_10_ *p-*values. Each SNP is plotted as a red dot, and the black line indicates the null hypothesis of no true association.

The average 25(OH)D concentrations (SD) of the cohorts ranged from 38.0 (17.2) nmol/l in EPIC-InterAct to 58.6 (23.9) nmol/l in the Ely study (Table 1). For total 25(OH)D level (Fig 2; Table 2), in the meta-analysis of 9 sets of GWASs among 120,618 participants, we identified 10 genetic loci. Of these 10 loci, we confirmed 6 previously discovered loci at *GC* (rs3755967, *p =* 2.48 × 10^−465^), *CYP2R1* (rs116970203, *p =* 1.19 × 10^−64^), *NADSYN1/DHCR7* (rs12785878, *p =* 5.60 × 10^−87^), *AMDHD1* (rs3213737, *p =* 2.05 × 10^−19^), *SEC23A* (rs8018720, *p =* 1.46 × 10^−10^), and *CYP24A1* (rs17216707, *p =* 1.61 × 10^−29^). Additionally, we identified 4 novel loci at *PADI1* (rs11203339, *p =* 4.64 × 10^−8^), *CRCT1* (rs7529325, *p =* 2.09 × 10^−9^), *UGT1A5* (rs17862870, *p =* 5.57 × 10^−9^), and *SULT2A1* (rs9304669, *p =* 4.53 × 10^−8^) (Table 2; S1–S3 Figs). The association of total 25(OH)D SNPs with each 25(OH)D metabolite is shown in S1 Table. In the UK Biobank, we replicated associations of all the above 10 SNPs, with *p-*values < 10^−33^ (S2 Table).

**Table 2:** Genetic loci identified in the genome-wide analyses for circulating 25(OH)D metabolites.

Circulating vitamin D	Gene	Role of the gene in vitamin D metabolism	Lead SNP	Chromosome	Position	Effect allele/other allele	EAF	Effect (beta)	Standard error	*p-*Value
Total 25(OH)D (*N* = 120,618)	*PADI1*^†^	Other	rs11203339	1	17560972	C/T	0.66	0.012	0.002	4.64 × 10^−08^
	*CRCT1*^†^	Other	rs7529325	1	152492634	A/T	0.08	0.030	0.005	2.09 × 10^−09^
	*UGT1A5*^†^	Other	rs17862870	2	234622742	G/A	0.92	0.021	0.004	5.57 × 10^−09^
	*GC*	Catabolism	rs3755967	4	72609398	C/T	0.71	0.106	0.002	2.48 × 10^−465^
	*CYP2R1*	Synthesis	rs116970203	11	14876718	G/A	0.97	0.381	0.022	1.19 × 10^−64^
	*NADSYN1/DHCR7*	Synthesis	rs12785878	11	71167449	T/G	0.75	0.044	0.002	5.60 × 10^−87^
	*AMDHD1*	Other	rs3213737	12	96379806	G/A	0.43	0.019	0.002	2.05 × 10^−19^
	*SEC23A*	Other	rs8018720	14	39556185	G/C	0.17	0.018	0.003	1.46 × 10^−10^
	*SULT2A1*^†^	Other	rs9304669	19	48384385	T/C	0.16	0.052	0.010	4.53 × 10^−08^
	*CYP24A1*	Catabolism	rs17216707	20	52732362	T/C	0.81	0.030	0.003	1.61 × 10^−29^
25(OH)D_3_ (*N* = 40,562)	*SHQ1*^†^	Other	rs13084927	3	72709792	C/A	0.83	0.055	0.010	1.94 × 10^−08^
	*GC*	Catabolism	rs4588	4	72618323	G/T	0.71	0.266	0.008	6.55 × 10^−266^
	*CYP2R1*	Synthesis	rs116970203	11	14876718	G/A	0.98	0.372	0.023	3.22 × 10^−60^
	*NADSYN1/DHCR7*	Synthesis	rs28364617	11	71159764	G/T	0.71	0.127	0.008	4.08 × 10^−59^
	*AMDHD1*	Other	rs3819817	12	96378771	C/T	0.45	0.058	0.007	3.59 × 10^−16^
	*SULT2A1*^†^	Other	rs9304669	19	48384385	T/C	0.16	0.054	0.010	1.27 × 10^−08^
	*CYP24A1*	Catabolism	rs17216707	20	52732362	T/C	0.80	0.074	0.009	1.09 × 10^−15^
C3-epi-25(OH)D_3_ (as a binary variable, *N* = 40,562)^‡^	*GC*	Catabolism	rs4588	4	72618323	G/T	0.71	0.194	0.016	2.48 × 10^−32^
	*NADSYN1/DHCR7*	Synthesis	rs28364617	11	71159764	G/T	0.72	0.131	0.017	1.95 × 10^−15^
	*SDR9C7*^†^	Other	rs11172066	12	57319491	T/A	0.14	0.166	0.021	8.08 × 10^−15^

Beta coefficients are in standard deviation (SD) unit per allele.

^†^Novel loci identified in the present genome-wide analyses.

^‡^C3-epi-25(OH)D_3_ was coded as binary variable: above versus below the lower limit of quantification (1 nmol/l).

25(OH)D, 25-hydroxyvitamin D; EAF, effect allele frequency; SNP, single nucleotide polymorphism.

Total 25(OH)D was not genetically correlated with T2D or glycaemic traits including HOMA-IR, HOMA-B, HbA1c, fasting insulin, fasting glucose, and 2-hour glucose (S3 Table). In the EPIC-Norfolk study, the variance explained by the 10 lead SNPs for total 25(OH)D was 3.95% (with F-statistic = 43.1), which was slightly higher than that explained (3.66%) by the 6 previously known loci (*GC*, *CYP2R1*, *NADSYN1*, *CYP24A1*, *AMDHD1*, and *SEC23A*). In the UK Biobank study, the variance explained by the 10 lead SNPs and the 6 previously known loci was 3.28% and 3.08%, respectively.

**Fig 3:**
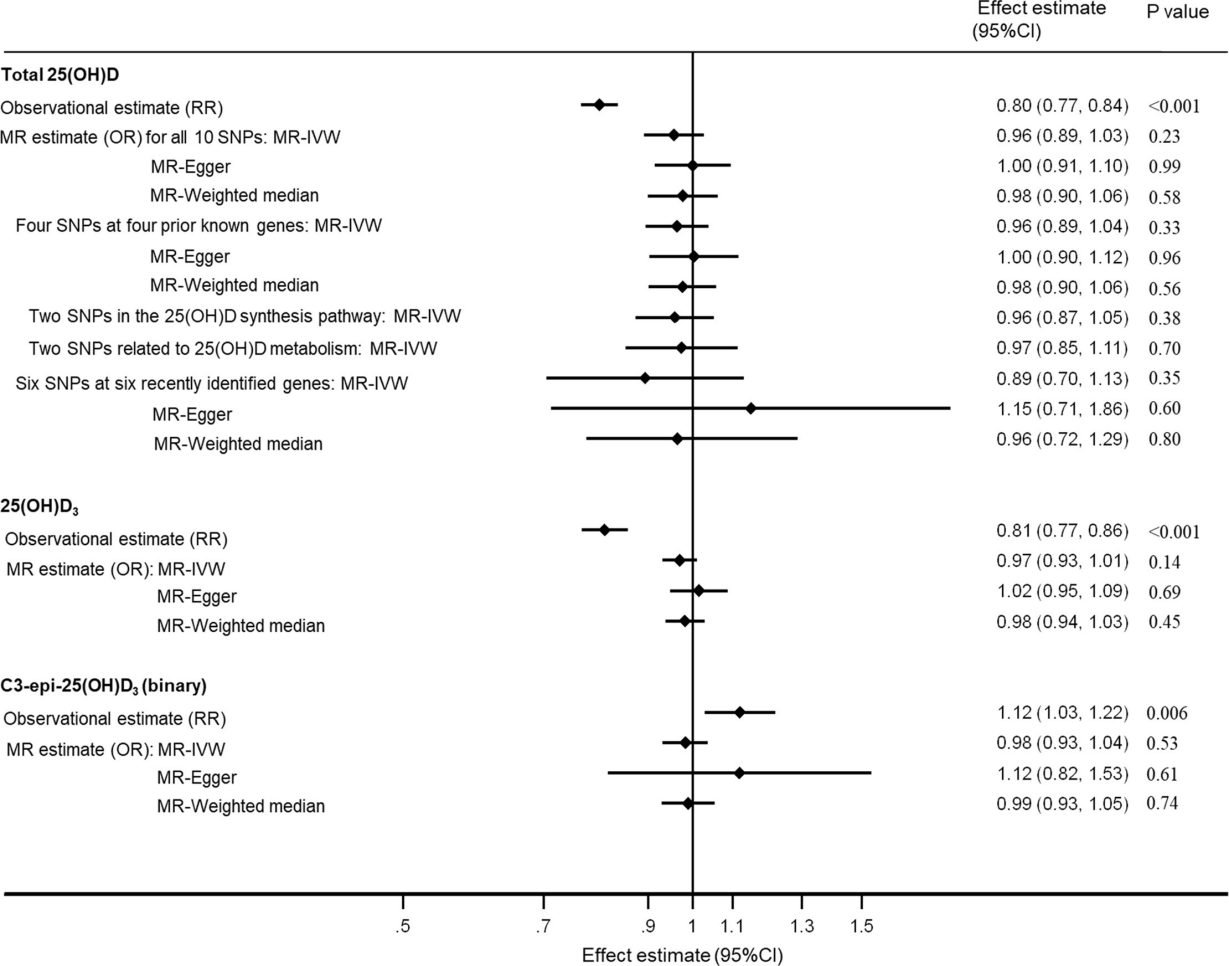
Association of 25(OH)D metabolites with type 2 diabetes from observational and MR analyses. Estimates (95% CIs) were scaled to represent RRs from observational analyses or ORs from MR per 1–standard deviation difference in each 25(OH)D metabolite, except for the binary C3-epi-25(OH)D_3_ variable (above versus below the lower limit of quantification). For the MR sensitivity analysis for total 25(OH)D, the 4 prior known genes are *GC*, *CYP2R1*, *NADSYN1/DHCR7*, and *CYP24A1*; the 25(OH)D synthesis pathway genes are *CYP2R1* and *NADSYN1/DHCR7*; the 25(OH)D metabolism genes are *GC* and *CYP24A1*; and the 6 recently identified genes are *PADI1*, *CRCT1*, *UGT1A5*, *AMDHD1*, *SEC23A*, and *SULT2A1*. For each analysis, 3 MR methods were used, including the IVW method, MR-Egger method, and weighted median method. For MR analyses based on 2 SNPs, the MR-Egger and weighted median methods could not be applied. For the observational estimate, the total number of type 2 diabetes cases and non-cases was 15,611 and 21,106, respectively, for total 25(OH)D, and 8,331 and 11,837, respectively, for 25(OH)D_3_ (or C3-epi-25(OH)D_3_). For the MR estimate of all 3 vitamin D variables, the total number of cases and non-cases was 80,983 and 842,909, respectively. 25(OH)D, 25-hydroxyvitamin D; CI, confidence interval; IVW, inverse-variance-weighted; MR, Mendelian randomisation; OR, odds ratio; RR, relative risk; SNP, single nucleotide polymorphism.

The observational analyses suggested that higher levels of 25(OH)D were associated with lower risk of T2D (per 1-SD RR: 0.80; 95% CI 0.77, 0.84; *p <* 0.001) (Fig 3). Further adjustment for BMI GRS (weighted or unweighted) and quadratic terms for BMI and waist-to-hip ratio had very little effect on the results (S4 Table).

In the MR analysis (Fig 3), we did not find evidence that genetically predicted higher total 25(OH)D was associated with T2D, with per 1-SD ORs of 0.96 (95% CI 0.89, 1.03; *p =* 0.23), 1.00 (95% CI 0.91, 1.10; *p =* 0.99), and 0.98 (95% CI 0.90, 1.06; *p =* 0.58) for the IVW, MR-Egger, and weighted median methods, respectively. Individual 25(OH)D genetic variants were not significantly associated with metabolic markers or lifestyle or demographic factors (S4 Fig). Testing the MR-Egger intercept (beta = −0.005; 95% CI −0.011, 0.001; *p =* 0.15) did not yield evidence of directional pleiotropy. MR-PRESSO, MR-RAPS, MRMix, and multivariable MR produced similar null results (S5 Fig). MR analyses suggested that total 25(OH)D was not significantly associated with any glycaemic trait (S5 Table).

### Individual 25(OH)D metabolites and T2D

For 25(OH)D_3_ (Fig 2; Table 2), we identified 7 novel genetic loci, of which one was a unique locus at *SHQ1* (rs13084927, *p =* 1.94 × 10^−8^), while the other 6 were already known to affect total 25(OH)D: *GC* (rs4588, *p =* 6.55 × 10^−266^), *CYP2R1* (rs116970203, *p =* 3.22 × 10^−60^), *NADSYN1/DHCR7* (rs28364617, *p =* 4.08 × 10^−59^), *AMDHD1* (rs3819817, *p =* 3.59 × 10^−16^), *SULT2A1* (rs9304669, *p =* 1.27 × 10^−8^), and *CYP24A1* (rs17216707, *p =* 1.09 × 10^−15^) (S6–S8 Figs).

For C3-epi-25(OH)D_3_, we identified 2 loci that overlapped with total 25(OH)D at *GC* (rs4588, *p =* 2.48 × 10^−32^) and *NADSYN1/DHCR7* (rs28364617, *p =* 1.95 × 10^−15^), and 1 unique locus at *SDR9C7* (rs11172066, *p =* 8.08 × 10^−15^) (Table 2; S9–S11 Figs).

Neither 25(OH)D_3_ nor C3-epi-25(OH)D_3_ was genetically correlated with T2D or glycaemic traits including HOMA-IR, HOMA-B, HbA1c, fasting insulin, fasting glucose, and 2-hour glucose (S4 Table). In the EPIC-Norfolk study, the variance explained by the lead SNPs was 4.58% for 25(OH)D_3_ (with F-statistic = 71.2) and 0.41% for C3-epi-25(OH)D_3_ (binary variable) (with F-statistic = 15.2).

In the non-genetic observational analyses, circulating 25(OH)D_3_ was inversely associated with T2D (per 1-SD RR: 0.81; 95% CI 0.77, 0.86; *p <* 0.001), and C3-epi-25(OH)D_3_ was positively associated with T2D (above versus below LLQ RR: 1.12; 95% CI 1.03, 1.22; *p =* 0.006) (Fig 3). Further adjustment for BMI GRS (weighted or unweighted) and quadratic terms for BMI and waist-to-hip ratio did not change the estimate for 25(OH)D_3_, while slightly strengthening the positive association for C3-epi-25(OH)D_3_ (S5 Table).

There was no evidence of pleiotropic associations of the genetic variants with metabolic markers or lifestyle or demographic factors (S12 and S13 Figs). Genetically predicted increases in 25(OH)D_3_ or high levels of C3-epi-25(OH)D_3_ (binary) were not associated with T2D (Fig 3). The findings did not change substantially in the analyses using other MR methods (S5 Fig) and in secondary analyses stratifying SNPs into different groups (S14 Fig). Testing the MR-Egger intercept did not yield evidence of directional pleiotropy for 25(OH)D_3_ (beta = −0.008; 95% CI −0.018, 0.002; *p =* 0.16) or C3-epi-25(OH)D_3_ (beta = −0.021; 95% CI −0.073, 0.03; *p =* 0.65). None of the genetically predicted variations of vitamin D metabolites were significantly associated with the glycaemic outcomes (S5 Table).

## Discussion

Our updated meta-analysis of GWASs for total 25(OH)D levels using data from 120,618 European-descent participants identified 4 novel genetic loci, in addition to 6 loci previously described [19]. The 4 novel loci were replicated in the UK Biobank study. With GWAS-identified lead SNPs as a genetic instrument, our MR analysis did not find evidence that total 25(OH)D was causally associated with T2D. To the best of our knowledge, the present study is the first GWAS discovery for 25(OH)D_3_, the major metabolite of circulating total 25(OH)D, identifying 7 genetic loci, with 1 unique locus and 6 loci overlapping with those for total 25(OH)D. Similarly, for C3-epi-25(OH)D_3_, we identified 1 unique locus associated with C3-epi-25(OH)D_3_. In the MR analysis, we did not find evidence supporting a causal association between T2D and 25(OH)D_3_ or C3-epi-25(OH)D_3_.

Our current finding of lack of evidence for a causal association of 25(OH)D with T2D in MR analysis is at variance with the strong inverse observational association between 25(OH)D and T2D [1,34]. This discrepancy was substantial, with non-overlapping confidence intervals (RR 0.80 [95% CI 0.77, 0.84] and 0.96 [95% CI 0.89, 1.03], respectively, for the observational and MR findings), and was not explained by detailed adjustment for the confounding effect of adiposity in the observational analysis. The reasons for the difference between the observational and MR findings remain unclear, but it may be that adjustment for adiposity, diet, and physical activity using a single imprecise measure of these variables at baseline only partially reduced the confounding effects of these variables. However, the current lack of evidence for a causal association between 25(OH)D levels and T2D is consistent with recent RCT evidence (D2d trial) of a lack of benefit from vitamin D supplementation for the prevention of T2D [6]. The D2d trial was conducted among individuals with prediabetes and thus at high risk for developing T2D [6]; prior RCTs among postmenopausal women or elderly people also had null findings [3,4], and the awaited results from the VITAL trial will be further informative.

Several prior MR analyses found inconsistent results [1,9–12]. In a MR analysis among 28,144 T2D cases and 76,344 controls, we previously used 4 prior GWAS-discovered genetic variants (at *GC*, *CYP2R1*, *DHCR7*, and *CYP24A1*) as genetic instruments, and found a null association between 25(OH)D and T2D (OR 1.01 [95% CI 0.75, 1.36] per 1-SD reduction in 25(OH)D) [1]. When we restricted our analysis to the 2 synthesis-related SNPs (at *DHCR7* and *CYP2R1*), we still had null findings [1], but this differed from other studies [11,12]. Afzal et al. examined variants in the 2 synthesis-related genes *DHCR7* and *CYP2R1* among 96,423 white Danish adults, reporting that per 20-nmol/l genetically determined reduction in plasma 25(OH)D there was an allelic effect for *DHCR7*, with an OR of 1.51 (95% CI 0.98, 2.33), but not for *CYP2R1* (OR 1.02 [95% CI 0.75–1.37]) [11]. The authors acknowledged that their results were weak and generated a hypothesis for a possible causal inverse association with endogenously synthesised 25(OH)D. Lu et al. detected a significant causal protective effect on T2D risk using 2 synthesis SNPs related to *DHCR7* and *CYP2R1* only when including Chinese and European populations in a meta-analysis [12]. The possibility of uncorrected population stratification by different ethnic groups [35] cannot be excluded, though the authors made attempts to reduce this possibility by analysing area-specific estimates and combining them using inverse-variance weighting. Notably, a recent Chinese study did not find a causal association between 25(OH)D and T2D [10].

Described for the first time, to the best of our knowledge, we found a genome-wide significant signal for 25(OH)D_3_ at *SHQ1*, which encodes H/ACA ribonucleoprotein assembly factor and has functions in the processing of ribosomal RNAs, modification of spliceosomal small nuclear RNAs, and stabilisation of telomerase [36]. In addition, we identified a novel locus at *SDR9C7* for C3-epi-25(OH)D_3_. *SDR9C7* encodes short chain dehydrogenase, whose relationship to the epimerisation of 25(OH)D_3_ to C3-epi-25(OH)D_3_ or other metabolites is unknown. Since the biological role of the epimerase is yet to be discovered [37], the present GWAS results may provide important insights for future investigation of mechanisms.

Although 25(OH)D_3_ is usually the major component of total 25(OH)D [2], the causal association of 25(OH)D_3_, on its own, with T2D risk has not previously been evaluated in MR analysis. Prior research mainly focused on total 25(OH)D as a biomarker of vitamin D status, which included a combination of 25(OH)D_2_ and 25(OH)D_3_, and, less frequently, C3-epi-25(OH)D_3_, the epimeric form of 25(OH)D_3_, depending on the assay methods. Blood 25(OH)D_3_ is derived both from diet and from biosynthesis in the skin upon exposure to sunlight, and 25(OH)D_2_ is mainly derived from diet, while C3-epi-25(OH)D_3_ is a metabolite of 25(OH)D_3_ via a C3 epimerisation process [37]. Traditional high-performance liquid chromatography, liquid chromatography–mass spectrometry, ELISA, and chemiluminescent immunoassay methods do not distinguish C3-epi-25(OH)D_3_ from 25(OH)D_3_ and thus include it within the definition of 25(OH)D_3_ [38]. Our recent study [2] suggested that blood 25(OH)D_3_ had an inverse association with T2D risk, while C3-epi-25(OH)D_3_ was positively associated. Therefore, these current findings are an important extension to previous research, by including MR analysis for T2D for total 25(OH)D as well as for 25(OH)D_3_ and C3-epi-25(OH)D_3_ separately, as the results for total 25(OH)D may be potentially confounded by other vitamin D metabolites.

There are several strengths of this study. To the best of our knowledge, this is the largest GWAS meta-analysis to date of total 25(OH)D (*N =* 120,618), and used the most comprehensive genetic instrument for the MR estimate for 25(OH)D and T2D risk (based on 10 SNPs versus the 4 or 2 used previously [1,11,12]). We replicated our total 25(OH)D GWAS results in the UK Biobank study, but were unable to evaluate genetic associations for 25(OH)D metabolites since these were not available in UK Biobank. In our MR analysis, we included 80,983 T2D cases, a larger sample than in the largest previous MR study (58,312 T2D cases) [12]. We performed a novel GWAS on individual vitamin D metabolites—25(OH)D_3_ and C3-epi-25(OH)D_3_—and used results from these in the MR analysis.

A limitation of this study is the lack of generalisability of our results from European populations to other populations of different ethnicities. Another limitation is that we combined studies with different study designs to maximise sample size and power for both MR analysis and observational analysis, even though definitions of endpoints (e.g., contributing to the degree of misclassification of T2D status) and exposure measurements (e.g., different assays for 25(OH)D measurement) were not standardised across these studies. In addition, we tested a linear causal association using the MR approach, but we cannot rule out a potential weak causal association among people with vitamin D deficiency or insufficiency. Furthermore, although we did not find convincing evidence for an association of these variants with a variety of metabolic markers and lifestyle and demographic factors, we cannot rule out the possibility that horizontal pleiotropy exists for the genetic variants used in the MR analysis. Finally, use of a binary exposure (C3-epi-25(OH)D_3_) in MR analysis can potentially lead to violation of the exclusion restriction assumption: The genetic variant can influence the outcome via the continuous risk factor even if the binary exposure does not change [39].

The public health implication of the present study, together with prior evidence of null findings from RCTs of vitamin D supplementation, is that focusing on population-wide supplementation to raise blood vitamin D levels is not likely to be an effective strategy for the prevention of T2D in European populations. However, our current limitations also indicate there are unresolved issues with important implications for further research. Next steps to address these issues include, but are not limited to, investigating the reasons for the discrepancy between the observational and MR findings, which likely reflect residual confounding as discussed above; including populations from different ethnic groups with varying distributions of 25(OH)D levels; and further increasing sample size and hence statistical power for conducting MR analysis of non-linear associations. The last of these would help to address whether there may be effects specifically in those who have hypovitaminosis D or are vitamin D deficient.

In summary, the present findings using MR do not support a causal association of 25(OH)D and related metabolites with T2D, despite the strong association seen in observational studies. The totality of the available evidence from our study and RCTs to date does not justify the use of vitamin D supplementation for the prevention of T2D.

## Supporting information

S1 STROBE Checklist STROBE checklist. (DOCX)

S1 Fig Regional plots of the genome-wide significant loci for total 25-hydroxyvitamin D. For each of the genetic loci, we used LocusZoom software to draw the regional association plot.
(TIF)

S2 Fig QQ plots by GWAS performed for total 25-hydroxyvitamin D. GWAS, genome-wide association study; QQ, quantile–quantile.
(TIF)

S3 Fig Association of novel identified genetic variants with total 25-hydroxyvitamin D in each GWAS. Effect estimate (95% confidence interval) of each forest plot represents the change (in standard deviation unit) in total 25-hydroxyvitamin D per allele of the corresponding genetic variant across participating cohorts. GWAS, genome-wide association study.
(TIF)

S4 Fig Association of total 25-hydroxyvitamin D genetic variants with metabolic markers and lifestyle and demographic factors. Effect estimate (95% confidence interval) of each forest plot represents the change in each trait per allele of the corresponding genetic variant. The summary statistics shown in the present figure were extracted from the PhenoScanner database (http://www.phenoscanner.medschl.cam.ac.uk/). We extracted the results with the largest sample size if results from multiple data sources were available in the PhenoScanner database. The corresponding databases in PhenoScanner were UK Biobank for body mass index and diastolic and systolic blood pressure, GIANT for waist-to-hip ratio (PMID: 25673412), GLGC for the 4 lipid traits (PMID: 24097068), MAGIC for the 6 glycaemic traits (PMID: 20081857), SSGAC for years of educational attainment (PMID: 27225129), and TAG for ever smoker (PMID: 20418890). *p <* 0.003 was considered statistically significant after correction for multiple testing within each genetic variant, and none of the results were significant. HbA1c, glycated haemoglobin; HOMA-B, homeostatic model assessment of beta cell function; HOMA-IR, homeostatic model assessment of insulin resistance.
(TIF)

S5 Fig Forest plots for the Mendelian randomisation analysis of 25-hydroxyvitamin D metabolites and type 2 diabetes. The Mendelian randomisation estimate is per 1-SD increase in vitamin D metabolite, except for the binary C3-epi-25(OH)D_3_ variable (above versus below the lower limit of quantification). None of the results show significant heterogeneity (*p =* 0.661 from Q-test) or directional horizontal pleiotropy (*p =* 0.153 from test of Egger intercept). For multivariable MR analysis, the result of total 25(OH)D or 25(OH)D_3_ was adjusted for the genetic variants of C3-epi-25(OH)D_3_, while the multivariable MR result of C3-epi-25(OH)D_3_ was adjusted for total 25(OH)D, as the definition of total 25(OH)D includes only 25(OH)D_3_ and 25(OH)D_2_, not C3-epi-25(OH)D_3_. For C3-epi-25(OH)D_3_, MR-PRESSO result was not available due to limited number of genetic variants.
(TIF)

S6 Fig Regional plots of the genome-wide significant loci for 25-hydroxyvitamin D_3_. For each of the genetic loci, we used LocusZoom software to draw the regional association plot.
(TIF)

S7 Fig QQ plots by GWAS performed for total 25-hydroxyvitamin D_3_. GWAS, genome-wide association study; QQ, quantile–quantile.
(TIF)

S8 Fig Association of novel identified genetic variants with 25-hydroxyvitamin D_3_ in each GWAS. Effect estimate (95% confidence interval) of each forest plot represents the change (in standard deviation unit) in total 25-hydroxyvitamin D_3_ per allele of the corresponding genetic variant across participating cohorts. GWAS, genome-wide association study.
(TIF)

S9 Fig Regional plots of the genome-wide significant loci for C3-epi-25-hydroxyvitamin D_3_. For each of the genetic loci, we used LocusZoom software to draw the regional association plot.
(TIF)

S10 Fig QQ plots by GWAS performed for C3-epi-25-hydroxyvitamin D_3_. GWAS, genome-wide association study; QQ, quantile–quantile.
(TIF)

S11 Fig Association of novel identified genetic variants with C3-epi-25-hydroxyvitamin D_3_ in each GWAS. Effect estimate (95% confidence interval) of each forest plot represents the change (log odds) in C3-epi-25-hydroxyvitamin D_3_ per allele of the corresponding genetic variant across participating cohorts. GWAS, genome-wide association study.
(TIF)

S12 Fig Association of 25-hydroxyvitamin D_3_ genetic variants with metabolic markers and lifestyle and demographic factors. The summary statistics shown in the present figure were extracted from the PhenoScanner database (http://www.phenoscanner.medschl.cam.ac.uk/). We extracted the results with the largest sample size if results from multiple data sources were available in the PhenoScanner database. The corresponding databases in PhenoScanner were UK Biobank for body mass index and diastolic and systolic blood pressure, GIANT for waist-to-hip ratio (PMID: 25673412), GLGC for the 4 lipid traits (PMID: 24097068), MAGIC for the 6 glycaemic traits (PMID: 20081857), SSGAC for years of educational attainment (PMID: 27225129), and TAG for ever smoker (PMID: 20418890). *p <* 0.003 was considered statistically significant after correction for multiple testing within each genetic variant, and none of the results were significant. HbA1c, glycated haemoglobin; HOMA-B, homeostatic model assessment of beta cell function; HOMA-IR, homeostatic model assessment of insulin resistance.
(TIF)

S13 Fig Association of C3-epi-25-hydroxyvitamin D_3_ genetic variants with metabolic markers and lifestyle and demographic factors. Effect estimate (95% confidence interval) of each forest plot represents the change in each trait per allele of the corresponding genetic variant. The summary statistics shown in the present figure were extracted from the PhenoScanner database (http://www.phenoscanner.medschl.cam.ac.uk/). We extracted the results with the largest sample size if results from multiple data sources were available in the PhenoScanner database. The corresponding databases in PhenoScanner were UK Biobank for body mass index and diastolic and systolic blood pressure, GIANT for waist-to-hip ratio (PMID: 25673412), GLGC for the 4 lipid traits (PMID: 24097068), MAGIC for the 6 glycaemic traits (PMID: 20081857), SSGAC for years of educational attainment (PMID: 27225129), and TAG for ever smoker (PMID: 20418890). *p <* 0.003 was considered statistically significant after correction for multiple testing within each genetic variant, and none of the results were significant. HbA1c, glycated haemoglobin; HOMA-B, homeostatic model assessment of beta cell function; HOMA-IR, homeostatic model assessment of insulin resistance.
(TIF)

S14 Fig Mendelian randomisation analysis of 25-hydroxyvitamin D metabolites and type 2 diabetes with stratification of single nucleotide polymorphisms. Mendelian randomisation (MR) estimate represents the association between a genetically predicted 1–standard deviation increase in 25-hydroxyvitamin D metabolites (except for the binary C3-epi-25(OH)D_3_ variable: above versus below the lower limit of quantification) and T2D risk. For 25(OH)D_3_, 4 prior known genes are *GC* (rs4588), *CYP2R1* (rs116970203), *NADSYN1/DHCR7* (rs28364617), and *CYP24A1* (rs17216707). Two genes in the 25(OH)D synthesis pathway are *CYP2R1* (rs116970203) and *NADSYN1/DHCR7* (rs28364617). Two genes related to 25(OH)D metabolism are *GC* (rs4588) and *CYP24A1* (rs17216707). Three genes, *SHQ1*, *AMDHD1*, and *SULT2A1*, were recently identified in the present GWAS meta-analysis or by another recent study. For C3-epi-25(OH)D_3_, we did sensitivity analysis stratified by the *SDR9C7* variant and the other variants (related to total 25(OH)D), as *SDR9C7* is a unique locus associated with C3-epi-25(OH)D_3_. The *SDR9C7* SNP was rs11172066. 25(OH)D, 25-hydroxyvitamin D; SNP, single nucleotide polymorphism.
(TIF)

S1 Table Association of GWAS-identified genetic variants with each of the 25-hydroxyvitamin D metabolites. (DOCX)

S2 Table Replication of the GWAS-identified variants for total 25-hydroxyvitamin D in the UK Biobank study. (DOCX)

S3 Table Genetic correlation of 25-hydroxyvitamin D metabolites with type 2 diabetes and glycaemic traits. (DOCX)

S4 Table Observational estimates of the association of 25-hydroxyvitamin D metabolites with incident type 2 diabetes in the EPIC-InterAct study. (DOCX)

S5 Table Mendelian randomisation analysis of 25-hydroxyvitamin D metabolites and glycaemic traits. (DOCX)

S1 Text Study protocol. (DOCX)

S2 Text Description of the participating studies in the present genome-wide association study for vitamin D metabolites. (DOCX)

## Abbreviations

25(OH)D: 25-hydroxyvitamin D
EPIC: European Prospective Investigation into Cancer and Nutrition
GRS: genetic risk score
GWAS: genome-wide association study
HbA1c: glycated haemoglobin
HOMA-B: homeostatic model assessment of beta cell function
HOMA-IR: homeostatic model assessment of insulin resistance
IVW: inverse-variance-weighted
LLQ: lower limit of quantification
MR: Mendelian randomisation
OR: odds ratio
RR: relative risk
RTC: randomised controlled trial
SD: standard deviation
SNP: single nucleotide polymorphism
T2D: type 2 diabetes
