# Supplementary figures and images for: The association between circulating 25-hydroxyvitamin D metabolites and type 2 diabetes in European populations: A meta-analysis and Mendelian randomisation analysis
Source: PLoS Med. 2020 Oct 16;17(10):e1003394. doi: 10.1371/journal.pmed.1003394 (PMC7567390; doi:10.1371/journal.pmed.1003394)

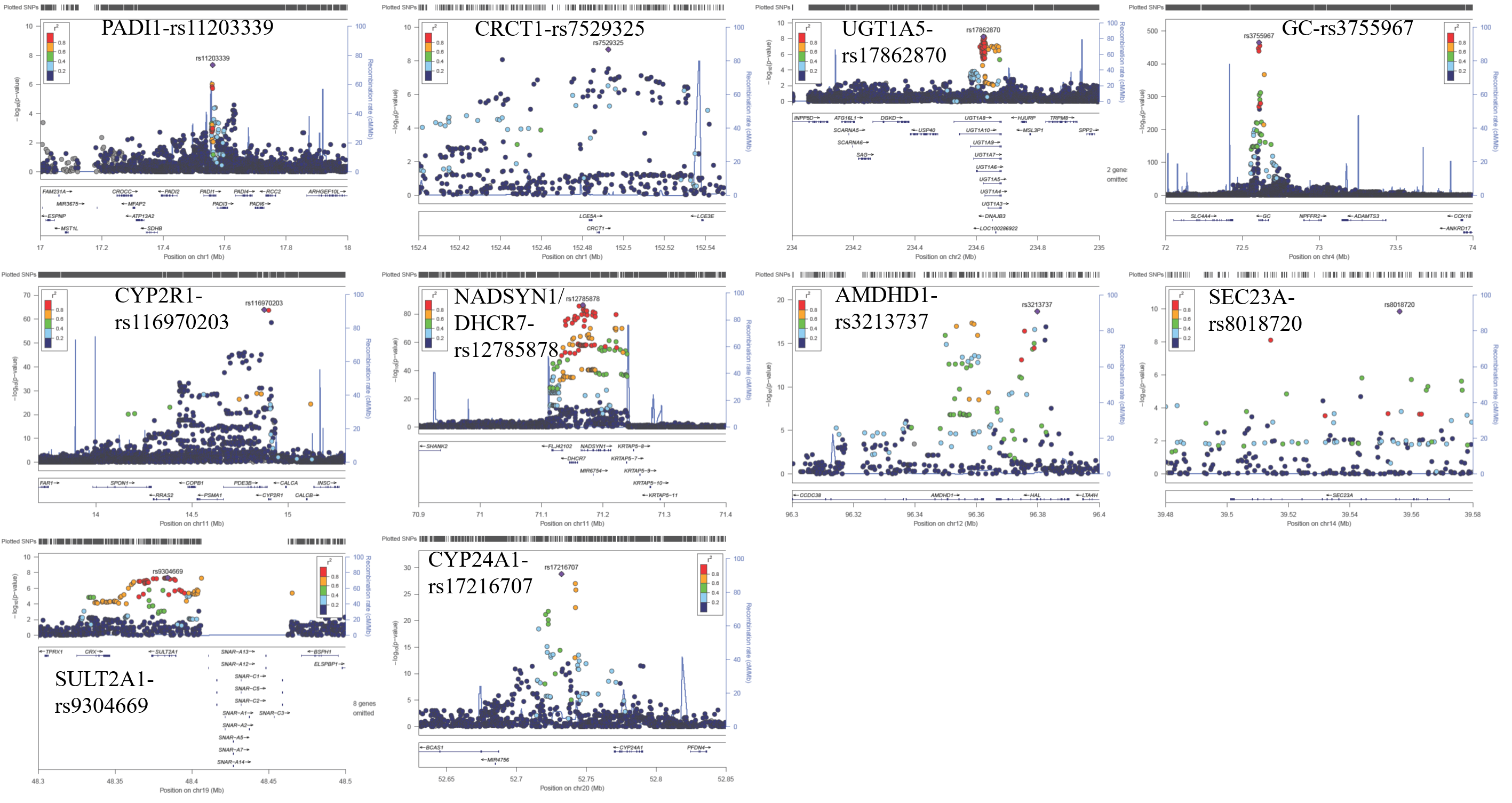

Supplement: S1 Fig — For each of the genetic loci, we used LocusZoom software to draw the regional association plot. (TIF) [file pmed.1003394.s002.tif]

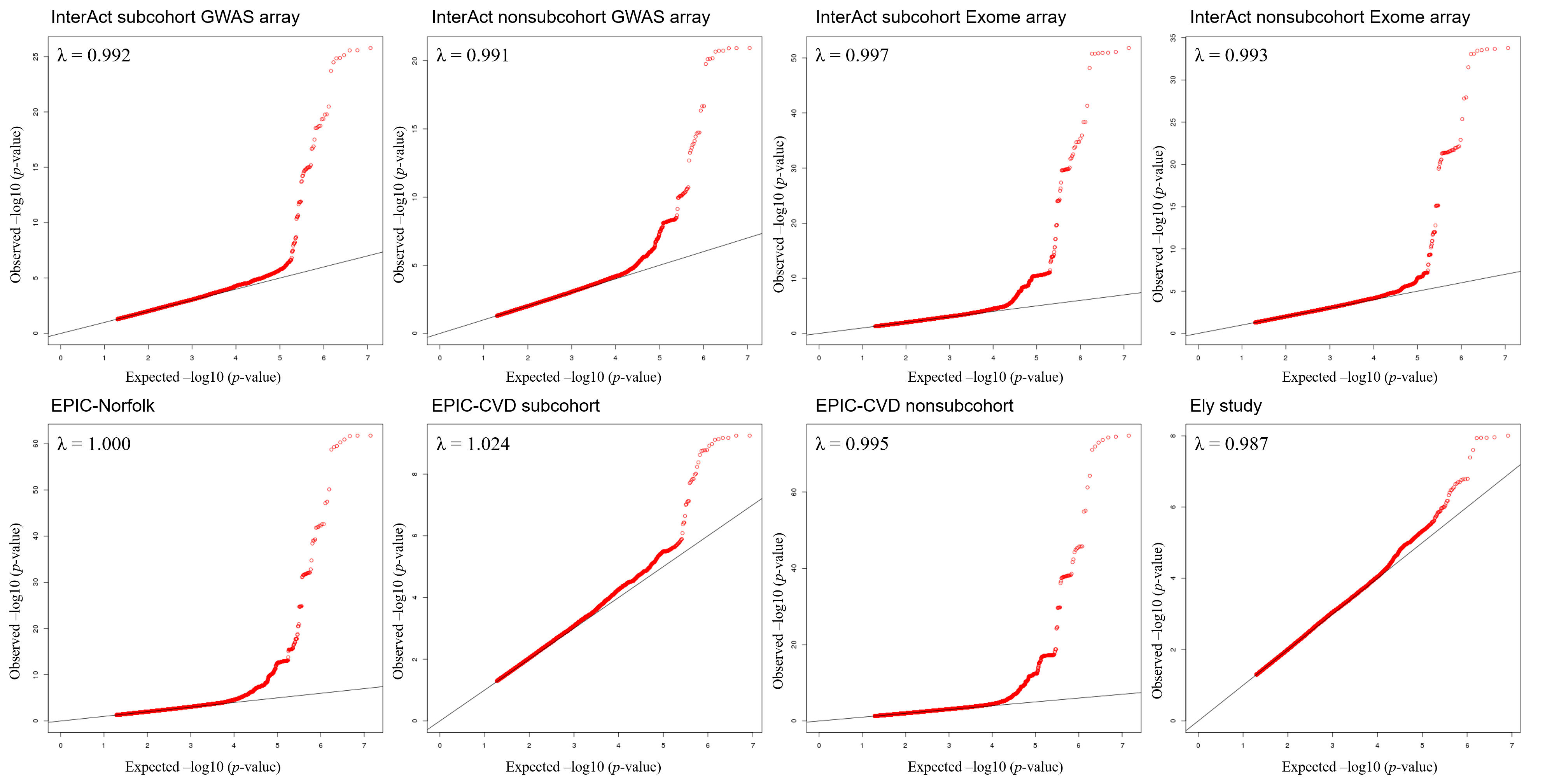

Supplement: S2 Fig — GWAS, genome-wide association study; QQ, quantile–quantile. (TIF) [file pmed.1003394.s003.tif]

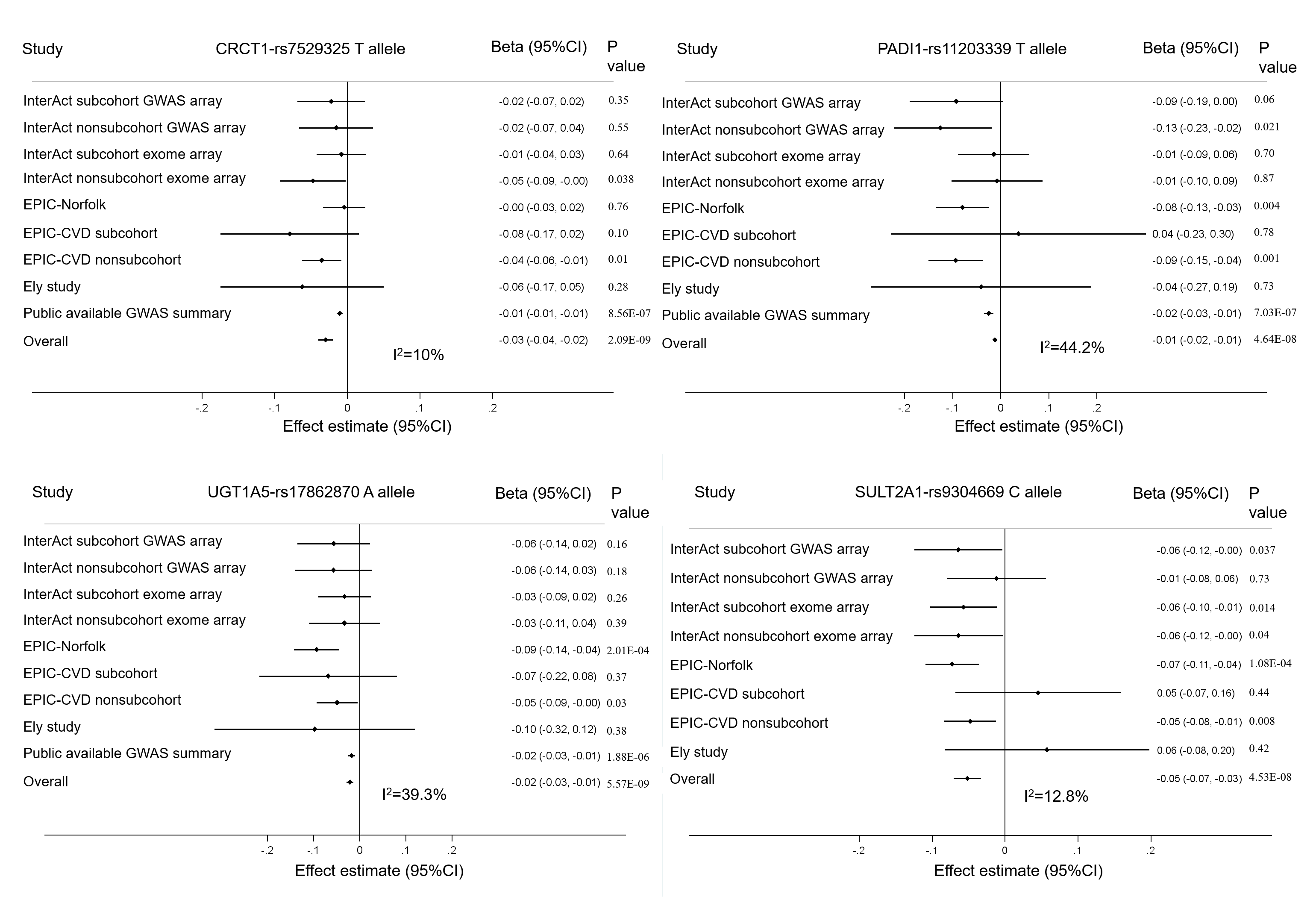

Supplement: S3 Fig — Effect estimate (95% confidence interval) of each forest plot represents the change (in standard deviation unit) in total 25-hydroxyvitamin D per allele of the corresponding genetic variant across participating cohorts. GWAS, genome-wide association study. (TIF) [file pmed.1003394.s004.tif]

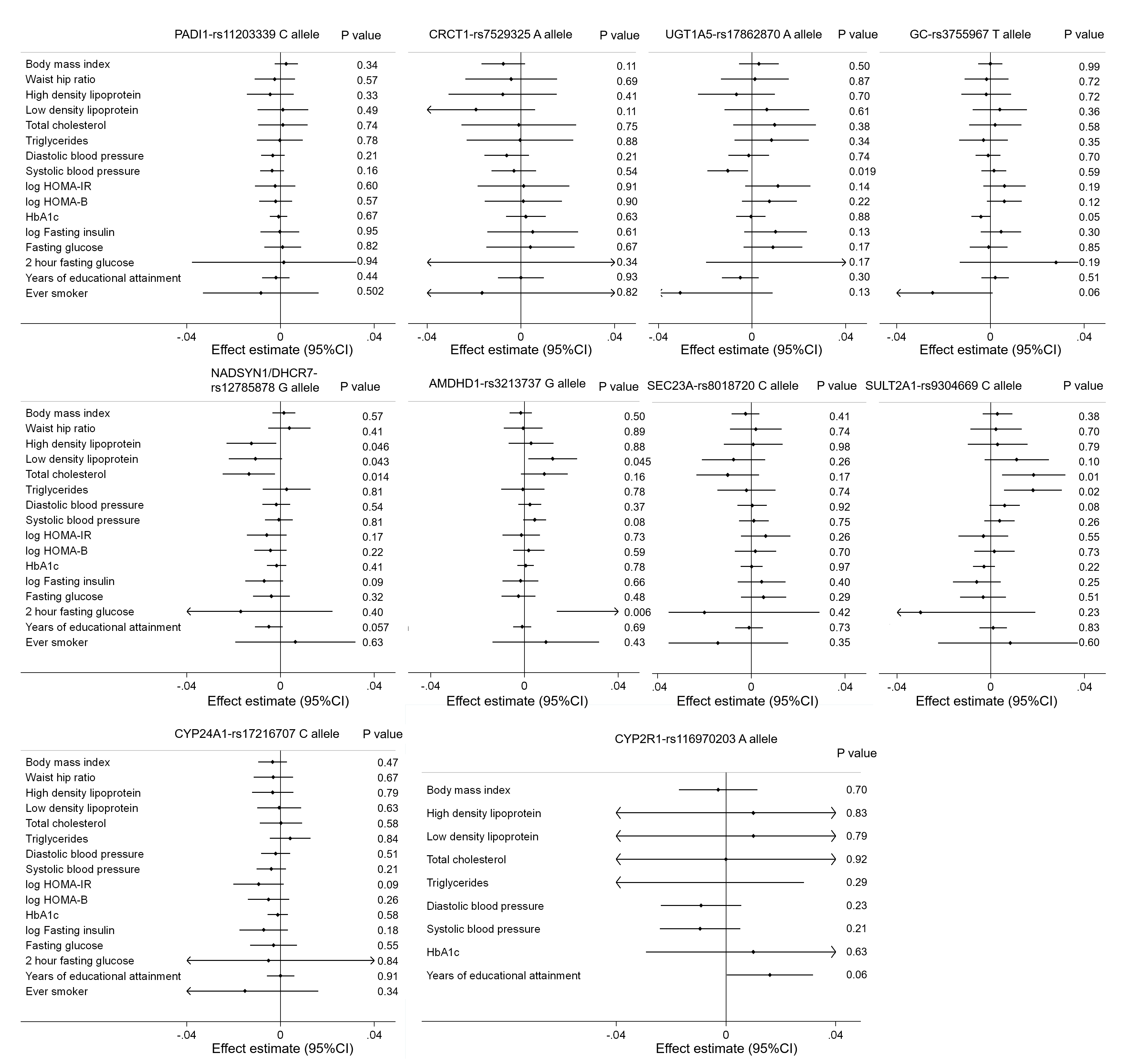

Supplement: S4 Fig — Effect estimate (95% confidence interval) of each forest plot represents the change in each trait per allele of the corresponding genetic variant. The summary statistics shown in the present figure were extracted from the PhenoScanner database (http://www.phenoscanner.medschl.cam.ac.uk/). We extracted the results with the largest sample size if results from multiple data sources were available in the PhenoScanner database. The corresponding databases in PhenoScanner were UK Biobank for body mass index and diastolic and systolic blood pressure, GIANT for waist-to-hip ratio (PMID: 25673412), GLGC for the 4 lipid traits (PMID: 24097068), MAGIC for the 6 glycaemic traits (PMID: 20081857), SSGAC for years of educational attainment (PMID: 27225129), and TAG for ever smoker (PMID: 20418890). p < 0.003 was considered statistically significant after correction for multiple testing within each genetic variant, and none of the results were significant. HbA1c, glycated haemoglobin; HOMA-B, homeostatic model assessment of beta cell function; HOMA-IR, homeostatic model assessment of insulin resistance. (TIF) [file pmed.1003394.s005.tif]

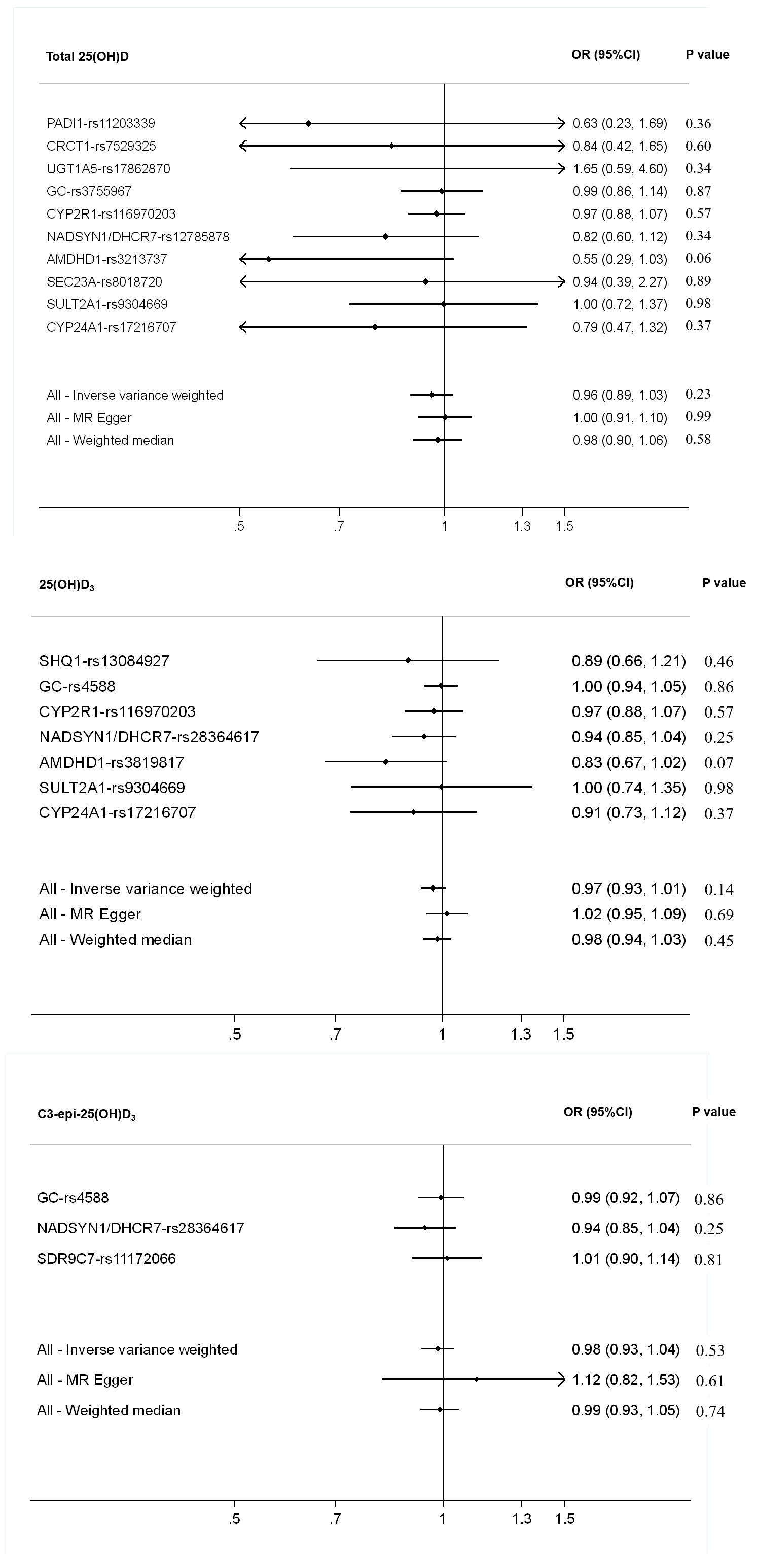

Supplement: S5 Fig — The Mendelian randomisation estimate is per 1-SD increase in vitamin D metabolite, except for the binary C3-epi-25(OH)D3 variable (above versus below the lower limit of quantification). None of the results show significant heterogeneity (p = 0.661 from Q-test) or directional horizontal pleiotropy (p = 0.153 from test of Egger intercept). For multivariable MR analysis, the result of total 25(OH)D or 25(OH)D3 was adjusted for the genetic variants of C3-epi-25(OH)D3, while the multivariable MR result of C3-epi-25(OH)D3 was adjusted for total 25(OH)D, as the definition of total 25(OH)D includes only 25(OH)D3 and 25(OH)D2, not C3-epi-25(OH)D3. For C3-epi-25(OH)D3, MR-PRESSO result was not available due to limited number of genetic variants. (TIF) [file pmed.1003394.s006.tif]

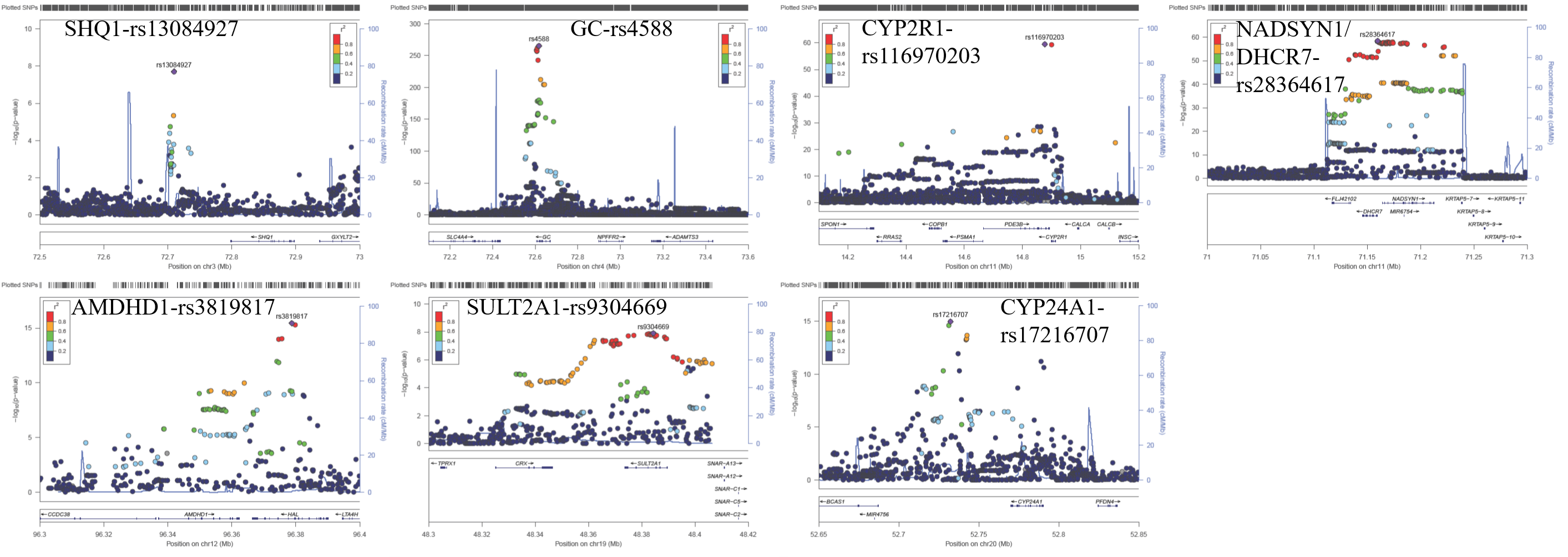

Supplement: S6 Fig — For each of the genetic loci, we used LocusZoom software to draw the regional association plot. (TIF) [file pmed.1003394.s007.tif]

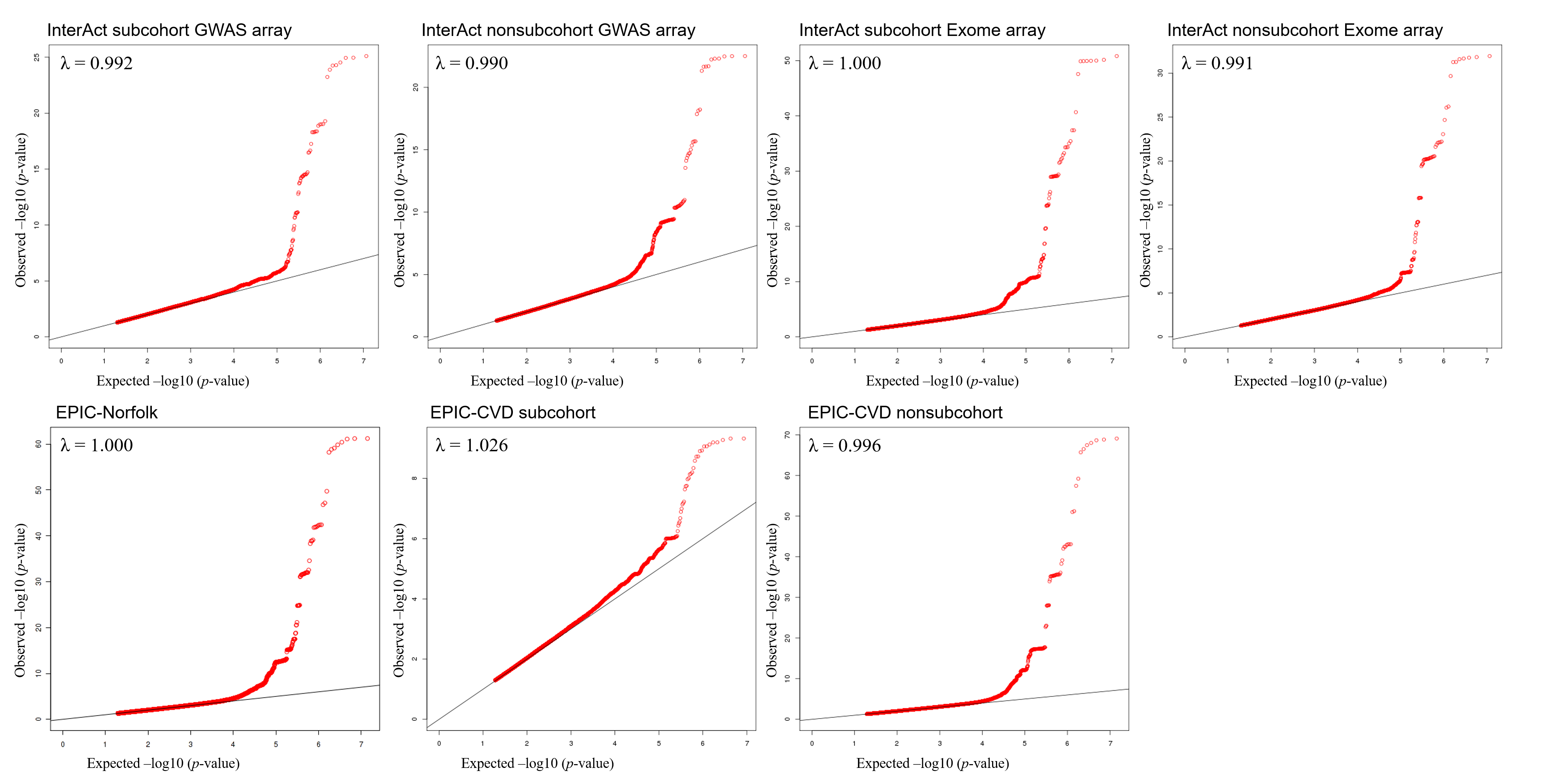

Supplement: S7 Fig — GWAS, genome-wide association study; QQ, quantile–quantile. (TIF) [file pmed.1003394.s008.tif]

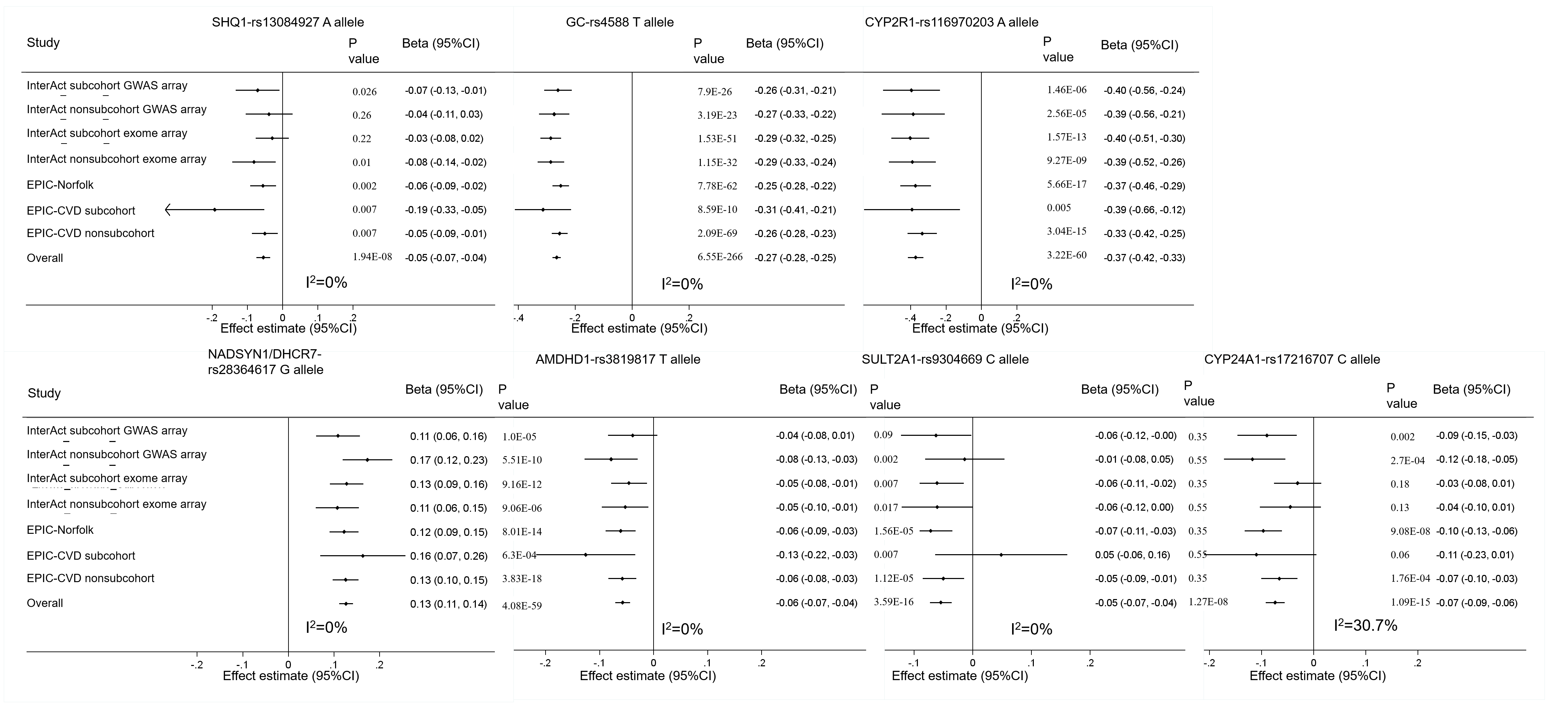

Supplement: S8 Fig — Effect estimate (95% confidence interval) of each forest plot represents the change (in standard deviation unit) in total 25-hydroxyvitamin D3 per allele of the corresponding genetic variant across participating cohorts. GWAS, genome-wide association study. (TIF) [file pmed.1003394.s009.tif]

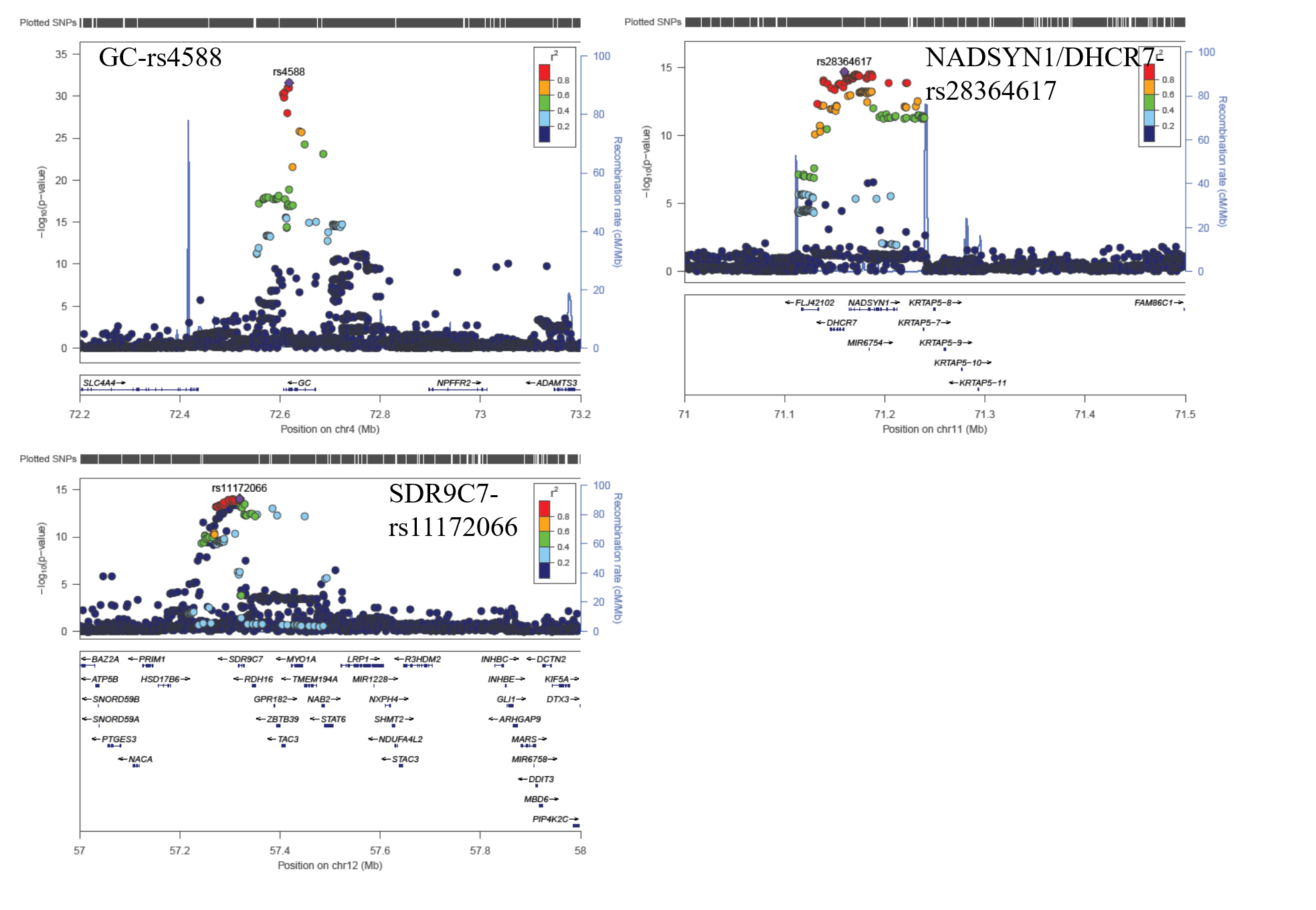

Supplement: S9 Fig — For each of the genetic loci, we used LocusZoom software to draw the regional association plot. (TIF) [file pmed.1003394.s010.tif]

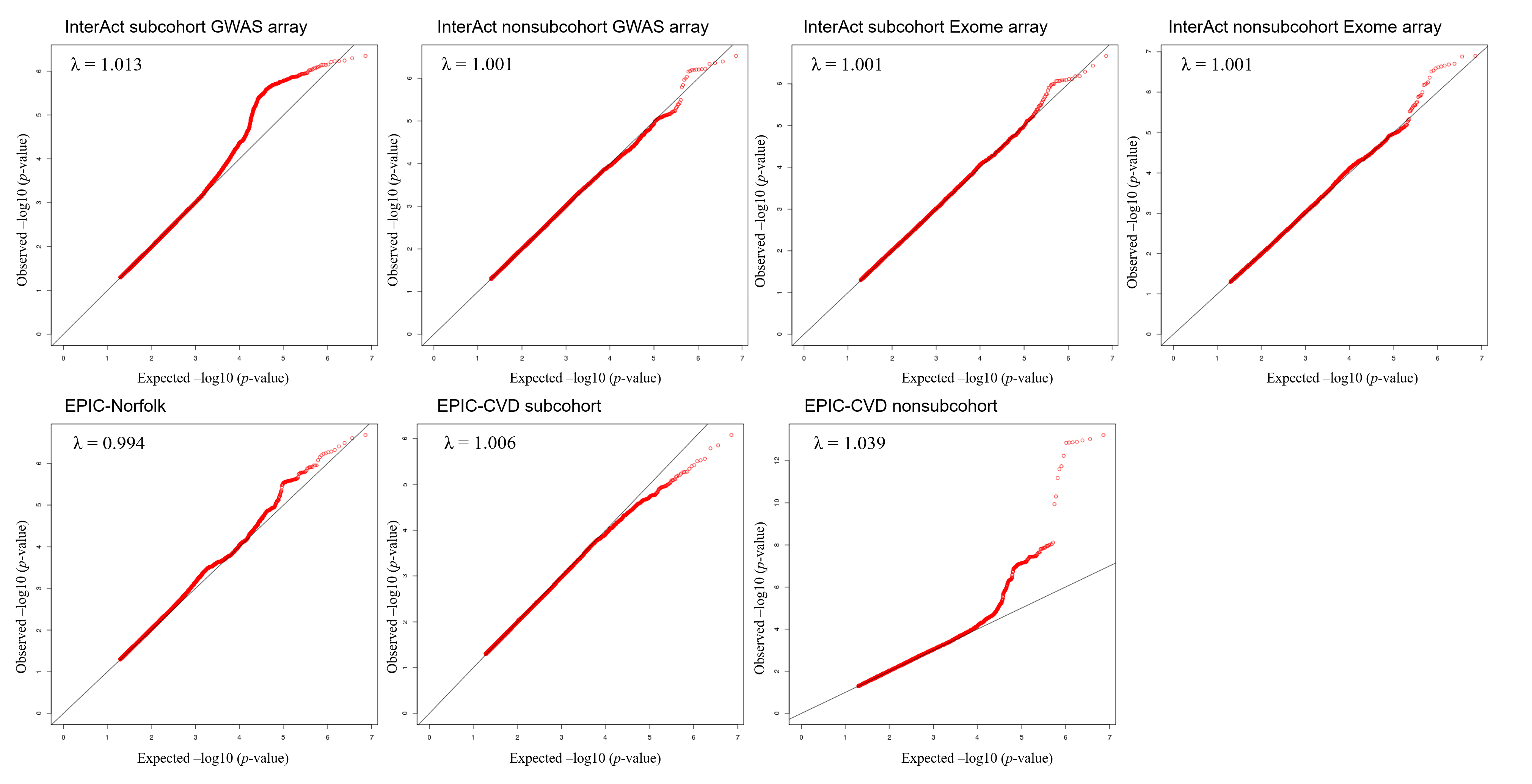

Supplement: S10 Fig — GWAS, genome-wide association study; QQ, quantile–quantile. (TIF) [file pmed.1003394.s011.tif]

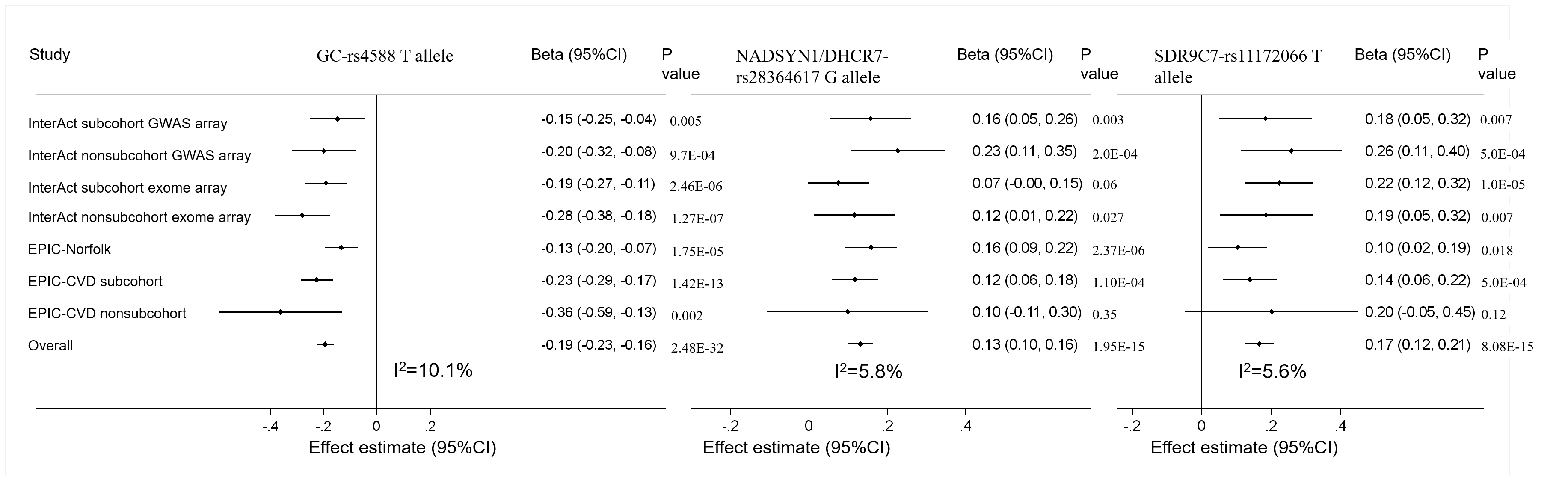

Supplement: S11 Fig — Effect estimate (95% confidence interval) of each forest plot represents the change (log odds) in C3-epi-25-hydroxyvitamin D3 per allele of the corresponding genetic variant across participating cohorts. GWAS, genome-wide association study. (TIF) [file pmed.1003394.s012.tif]

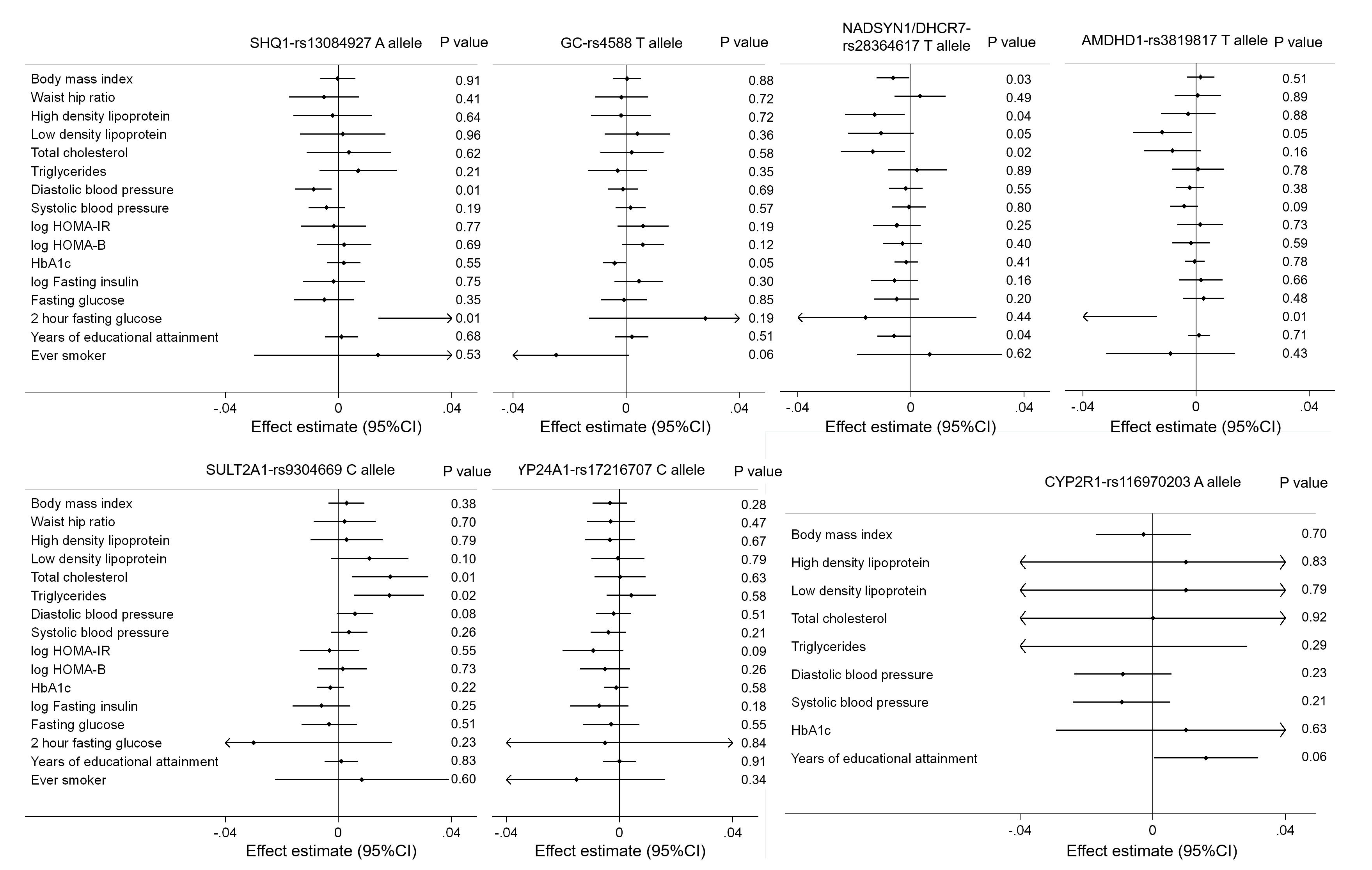

Supplement: S12 Fig — The summary statistics shown in the present figure were extracted from the PhenoScanner database (http://www.phenoscanner.medschl.cam.ac.uk/). We extracted the results with the largest sample size if results from multiple data sources were available in the PhenoScanner database. The corresponding databases in PhenoScanner were UK Biobank for body mass index and diastolic and systolic blood pressure, GIANT for waist-to-hip ratio (PMID: 25673412), GLGC for the 4 lipid traits (PMID: 24097068), MAGIC for the 6 glycaemic traits (PMID: 20081857), SSGAC for years of educational attainment (PMID: 27225129), and TAG for ever smoker (PMID: 20418890). p < 0.003 was considered statistically significant after correction for multiple testing within each genetic variant, and none of the results were significant. HbA1c, glycated haemoglobin; HOMA-B, homeostatic model assessment of beta cell function; HOMA-IR, homeostatic model assessment of insulin resistance. (TIF) [file pmed.1003394.s013.tif]

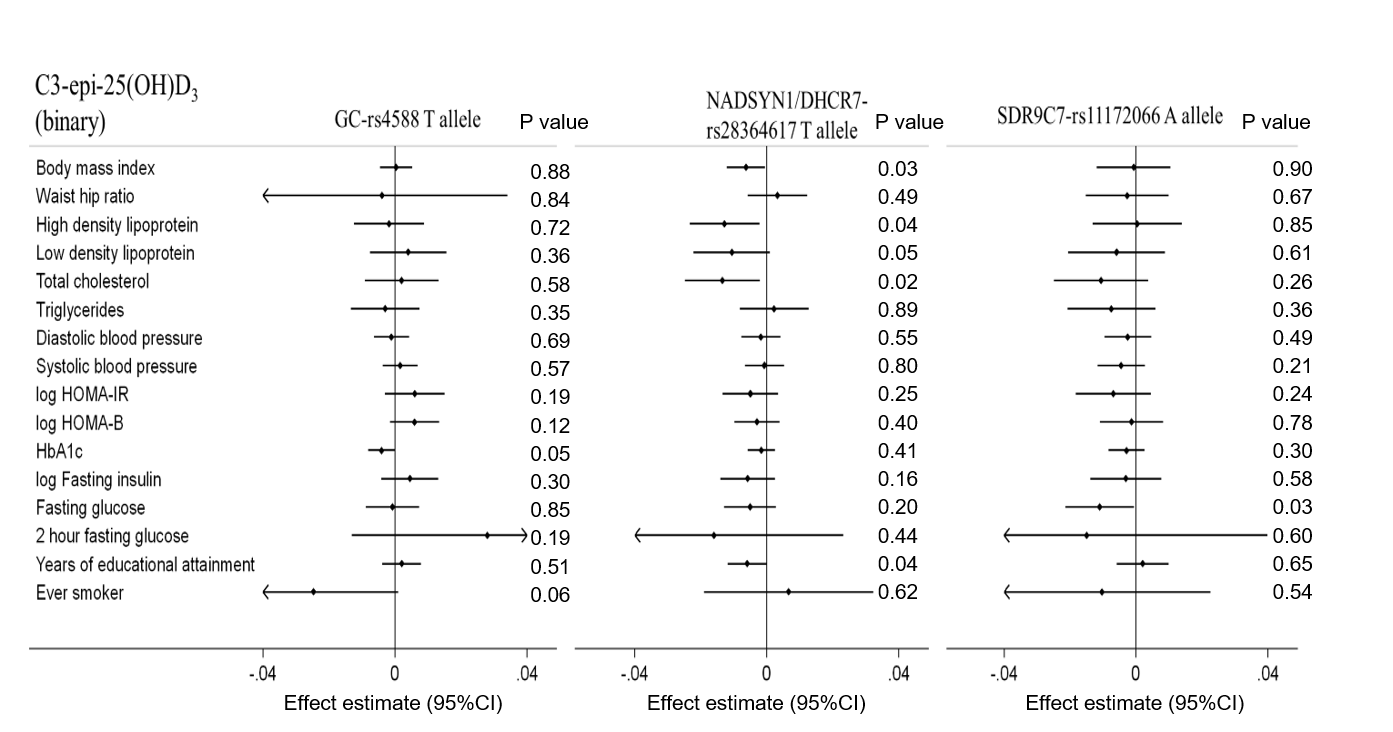

Supplement: S13 Fig — Effect estimate (95% confidence interval) of each forest plot represents the change in each trait per allele of the corresponding genetic variant. The summary statistics shown in the present figure were extracted from the PhenoScanner database (http://www.phenoscanner.medschl.cam.ac.uk/). We extracted the results with the largest sample size if results from multiple data sources were available in the PhenoScanner database. The corresponding databases in PhenoScanner were UK Biobank for body mass index and diastolic and systolic blood pressure, GIANT for waist-to-hip ratio (PMID: 25673412), GLGC for the 4 lipid traits (PMID: 24097068), MAGIC for the 6 glycaemic traits (PMID: 20081857), SSGAC for years of educational attainment (PMID: 27225129), and TAG for ever smoker (PMID: 20418890). p < 0.003 was considered statistically significant after correction for multiple testing within each genetic variant, and none of the results were significant. HbA1c, glycated haemoglobin; HOMA-B, homeostatic model assessment of beta cell function; HOMA-IR, homeostatic model assessment of insulin resistance. (TIF) [file pmed.1003394.s014.tif]

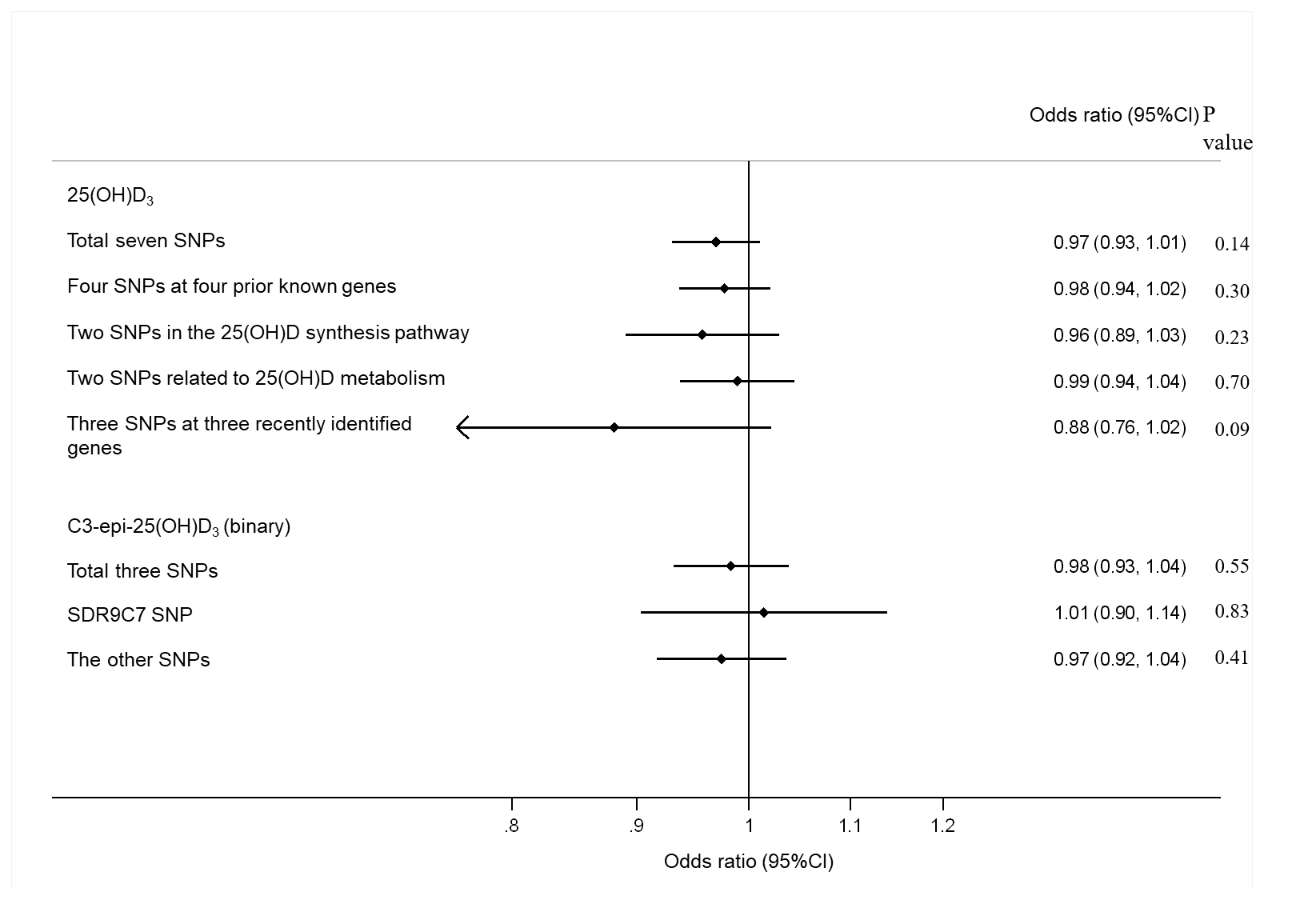

Supplement: S14 Fig — Mendelian randomisation (MR) estimate represents the association between a genetically predicted 1–standard deviation increase in 25-hydroxyvitamin D metabolites (except for the binary C3-epi-25(OH)D3 variable: above versus below the lower limit of quantification) and T2D risk. For 25(OH)D3, 4 prior known genes are GC (rs4588), CYP2R1 (rs116970203), NADSYN1/DHCR7 (rs28364617), and CYP24A1 (rs17216707). Two genes in the 25(OH)D synthesis pathway are CYP2R1 (rs116970203) and NADSYN1/DHCR7 (rs28364617). Two genes related to 25(OH)D metabolism are GC (rs4588) and CYP24A1 (rs17216707). Three genes, SHQ1, AMDHD1, and SULT2A1, were recently identified in the present GWAS meta-analysis or by another recent study. For C3-epi-25(OH)D3, we did sensitivity analysis stratified by the SDR9C7 variant and the other variants (related to total 25(OH)D), as SDR9C7 is a unique locus associated with C3-epi-25(OH)D3. The SDR9C7 SNP was rs11172066. 25(OH)D, 25-hydroxyvitamin D; SNP, single nucleotide polymorphism. (TIF) [file pmed.1003394.s015.tif]
