# Supplementary material for: The association between circulating 25-hydroxyvitamin D metabolites and type 2 diabetes in European populations: A meta-analysis and Mendelian randomisation analysis
Source: PLoS Med. 2020 Oct 16;17(10):e1003394. doi: 10.1371/journal.pmed.1003394 (PMC7567390; doi:10.1371/journal.pmed.1003394)
Supplement: S1 Table — (DOCX) [file pmed.1003394.s016.docx]

**S1 Table. Association of GWAS-identified genetic variants with each of the 25-hydroxyvitamin D metabolites***

| Gene-rsID | INFO score† | Effect allele/other allele | Total 25(OH)D | | | 25(OH)D_3_ | | | C3-epi-25(OH)D3 (binary) | | |
| --- | --- | --- | --- | --- | --- | --- | --- | --- | --- | --- | --- |
|  |  |  | Beta (95%CIs) | Standard Error | *p* value | Beta (95%CIs) | Standard Error | *p* value | Beta (95%CIs) | Standard Error | *p* value |
| **Total 25(OH)D** |  |  |  |  |  |  |  |  |  |  |  |
| *PADI1*-rs11203339 | 0.992 | C/T | 0.012 (0.008,0.016) | 0.002 | 4.64×10^-08^ | 0.023 (0.009,0.037) | 0.007 | 0.002 | 0.047 (0.018,0.076) | 0.015 | 0.002 |
| *CRCT1*-rs7529325 | 0.946 | A/T | 0.030 (0.02,0.04) | 0.005 | 2.09×10^-09^ | 0.07 (0.041,0.099) | 0.015 | 3.54×10^-06^ | 0.078 (0.015,0.141) | 0.032 | 0.013 |
| *UGT1A5*-rs17862870 | 1 | G/A | 0.021 (0.013,0.029) | 0.004 | 5.57×10^-09^ | 0.056 (0.032,0.08) | 0.012 | 5.75×10^-06^ | 0.063 (0.012,0.114) | 0.026 | 0.015 |
| *GC*-rs3755967 | 0.999 | C/T | 0.106 (0.102,0.11) | 0.002 | 2.48×10^-465^ | 0.264 (0.248,0.28) | 0.008 | 2.15×10^-262^ | 0.191 (0.16,0.222) | 0.016 | 3.01×10^-31^ |
| *CYP2R1*-rs116970203 | 0.988 | G/A | 0.381 (0.338,0.424) | 0.022 | 1.19×10^-64^ | 0.372 (0.327,0.417) | 0.023 | 3.22×10^-60^ | 0.207 (0.111,0.303) | 0.049 | 2.29×10^-05^ |
| *NADSYN1/DHCR7*-rs12785878 | 1 | T/G | 0.044 (0.04,0.048) | 0.002 | 5.60×10^-87^ | 0.125 (0.109,0.141) | 0.008 | 2.18×10^-58^ | 0.129 (0.096,0.162) | 0.017 | 5.29×10^-15^ |
| *AMDHD1*-rs3213737 | 0.995 | G/A | 0.019 (0.015,0.023) | 0.002 | 2.05×10^-19^ | 0.057 (0.043,0.071) | 0.007 | 4.79×10^-16^ | 0.056 (0.027,0.085) | 0.015 | 1.45×10^-04^ |
| *SEC23A*-rs8018720 | 1 | G/C | 0.018 (0.012,0.024) | 0.003 | 1.46×10^-10^ | 0.032 (0.014,0.05) | 0.009 | 6.67×10^-04^ | -0.012 (-0.051,0.027) | 0.020 | 0.558 |
| *SULT2A1*-rs9304669 | 0.999 | T/C | 0.052 (0.032,0.072) | 0.010 | 4.53×10^-08^ | 0.054 (0.034,0.074) | 0.010 | 1.27×10^-08^ | 0.018 (-0.021,0.057) | 0.020 | 0.360 |
| *CYP24A1*-rs17216707 | 0.963 | T/C | 0.03 (0.024,0.036) | 0.003 | 1.61×10^-29^ | 0.074 (0.056,0.092) | 0.009 | 1.09×10^-15^ | 0.016 (-0.021,0.053) | 0.019 | 0.397 |
| **25(OH)D_3_** |  |  |  |  |  |  |  |  |  |  |  |
| *SHQ1*-rs13084927 | 0.97 | C/A | 0.007 (0.001,0.013) | 0.003 | 0.020 | 0.055 (0.035,0.075) | 0.010 | 1.94×10^-08^ | 0.04 (-0.001,0.081) | 0.021 | 0.053 |
| *GC*-rs4588 | 1 | G/T | 0.271 (0.255,0.287) | 0.008 | 2.84×10^-280^ | 0.266 (0.25,0.282) | 0.008 | 6.55×10^-266^ | 0.194 (0.163,0.225) | 0.016 | 2.48×10^-32^ |
| *CYP2R1*-rs116970203 | 0.988 | G/A | 0.381 (0.338,0.424) | 0.022 | 1.19×10^-64^ | 0.372 (0.327,0.417) | 0.023 | 3.22×10^-60^ | 0.207 (0.111,0.303) | 0.049 | 2.29×10^-05^ |
| *NADSYN1/DHCR7*-rs28364617 | 0.997 | G/T | 0.126 (0.11,0.142) | 0.008 | 1.64×10^-59^ | 0.127 (0.111,0.143) | 0.008 | 4.08×10^-59^ | 0.131 (0.098,0.164) | 0.017 | 1.95×10^-15^ |
| *AMDHD1*-rs3819817 | 0.995 | C/T | 0.055 (0.041,0.069) | 0.007 | 3.11×10^-15^ | 0.058 (0.044,0.072) | 0.007 | 3.59×10^-16^ | 0.057 (0.028,0.086) | 0.015 | 1.13×10^-04^ |
| *SULT2A1*-rs9304669 | 0.999 | T/C | 0.052 (0.032,0.072) | 0.010 | 4.53×10^-08^ | 0.054 (0.034,0.074) | 0.010 | 1.27×10^-08^ | 0.018 (-0.021,0.057) | 0.020 | 0.360 |
| *CYP24A1*-rs17216707 | 0.963 | T/C | 0.03 (0.024,0.036) | 0.003 | 1.61×10^-29^ | 0.074 (0.056,0.092) | 0.009 | 1.09×10^-15^ | 0.016 (-0.021,0.053) | 0.019 | 0.397 |
| **C3-epi-25(OH)D_3_ (as a binary variable)** |  |  |  |  |  |  |  |  |  |  |  |
| *GC*-rs4588 | 1 | G/T | 0.271 (0.255,0.287) | 0.008 | 2.84×10^-280^ | 0.266 (0.25,0.282) | 0.008 | 6.55×10^-266^ | 0.194 (0.163,0.225) | 0.016 | 2.48×10^-32^ |
| *NADSYN1/DHCR7*-rs28364617 | 0.997 | G/T | 0.126 (0.11,0.142) | 0.008 | 1.64×10^-59^ | 0.127 (0.111,0.143) | 0.008 | 4.08×10^-59^ | 0.131 (0.098,0.164) | 0.017 | 1.95×10^-15^ |
| *SDR9C7*-rs11172066 | 0.996 | T/A | -0.001 (-0.007,0.005) | 0.003 | 0.883 | 0.01 (-0.01,0.03) | 0.010 | 0.315 | 0.166 (0.125,0.207) | 0.021 | 8.08×10^-15^ |

* GWAS, genome-wide association study; 25(OH)D, 25-hydroxyvitamin D. †imputation info score for all the identified genetic variants are very high (>0.9) across different studies, and we list those from EPIC-Norfolk here as an example.
