# Supplementary material for: The association between circulating 25-hydroxyvitamin D metabolites and type 2 diabetes in European populations: A meta-analysis and Mendelian randomisation analysis
Source: PLoS Med. 2020 Oct 16;17(10):e1003394. doi: 10.1371/journal.pmed.1003394 (PMC7567390; doi:10.1371/journal.pmed.1003394)
Supplement: S2 Table — (DOCX) [file pmed.1003394.s017.docx]

**S2 Table. Replication of the GWAS-identified variants for total 25-hydroxyvitamin D in the UK Biobank study***

| Gene | SNP | Chromosome | Position | Effect allele | Other allele | EAF | Beta (95%CIs) | Standard Error | *p* value | Estimates from the discovery* | |
| --- | --- | --- | --- | --- | --- | --- | --- | --- | --- | --- | --- |
|  |  |  |  |  |  |  |  |  |  | Effect (Beta) | Standard Error |
| *PADI1*† | rs11203339 | 1 | 17560972 | C | T | 0.65 | 0.021 (0.017,0.025) | 0.002 | 3.10×10^-20^ | 0.012 | 0.002 |
| *CRCT1*† | rs7529325 | 1 | 152492634 | A | T | 0.07 | 0.047 (0.039,0.055) | 0.004 | 6.80×10^-29^ | 0.030 | 0.005 |
| *UGT1A5*† | rs17862870 | 2 | 234622742 | G | A | 0.92 | 0.047 (0.039,0.055) | 0.004 | 3.00×10^-33^ | 0.021 | 0.004 |
| *GC* | rs3755967 | 4 | 72609398 | C | T | 0.71 | 0.199 (0.195,0.203) | 0.002 | 4.9×10^-1558^ | 0.106 | 0.002 |
| *CYP2R1* | rs116970203 | 11 | 14876718 | G | A | 0.97 | 0.376 (0.362,0.39) | 0.007 | 6.5×10^-723^ | 0.381 | 0.022 |
| *NADSYN1/DHCR7* | rs12785878 | 11 | 71167449 | T | G | 0.21 | 0.118 (0.112,0.124) | 0.003 | 9.7×10^-444^ | 0.044 | 0.002 |
| *AMDHD1* | rs3213737 | 12 | 96379806 | G | A | 0.42 | 0.043 (0.039,0.047) | 0.002 | 4.10×10^-88^ | 0.019 | 0.002 |
| *SEC23A* | rs8018720 | 14 | 39556185 | G | C | 0.18 | 0.034 (0.028,0.04) | 0.003 | 2.80×10^-34^ | 0.018 | 0.003 |
| *SULT2A1*† | rs9304669 | 19 | 48384385 | T | C | 0.17 | 0.065 (0.059,0.071) | 0.003 | 5.40×10^-113^ | 0.052 | 0.010 |
| *CYP24A1* | rs17216707 | 20 | 52732362 | T | C | 0.81 | 0.039 (0.033,0.045) | 0.003 | 8.60×10^-44^ | 0.030 | 0.003 |

* GWAS, genome-wide association study; EAF, effect allele frequency; SNP, single nucleotide polymorphism; Beta coefficients are in standard deviation (SD) unit per allele. Estimates from the discovery dataset are presented in Supplemental Table 2.

†novel loci identified in the present genome-wide analyses.
