# Supplementary material for: The association between circulating 25-hydroxyvitamin D metabolites and type 2 diabetes in European populations: A meta-analysis and Mendelian randomisation analysis
Source: PLoS Med. 2020 Oct 16;17(10):e1003394. doi: 10.1371/journal.pmed.1003394 (PMC7567390; doi:10.1371/journal.pmed.1003394)
Supplement: S3 Table — (DOCX) [file pmed.1003394.s018.docx]

**S3 Table. Genetic correlation of 25-hydroxyvitamin D metabolites with type 2 diabetes and glycaemic traits**

|  | Total 25(OH)D | | | 25(OH)D_3_ | | | C3-epi-25(OH)D_3_ | | |
| --- | --- | --- | --- | --- | --- | --- | --- | --- | --- |
|  | r (95%CIs)* | se | *p value* | r(95%CIs)* | se | *p value* | r(95%CIs)* | se | *p value* |
| Type 2 Diabetes | -0.07 (-0.186,0.046) | 0.059 | 0.236 | -0.112 (-0.263,0.039) | 0.077 | 0.149 | -0.141 (-0.429,0.147) | 0.147 | 0.339 |
| HOMA-IR | 0.039 (-0.128,0.206) | 0.085 | 0.647 | -0.046 (-0.266,0.174) | 0.112 | 0.684 | 0.037 (-0.435,0.509) | 0.241 | 0.879 |
| HOMA-B | -0.004 (-0.149,0.141) | 0.074 | 0.955 | -0.051 (-0.241,0.139) | 0.097 | 0.598 | -0.091 (-0.503,0.321) | 0.210 | 0.664 |
| HbA1C | 0.059 (-0.086,0.204) | 0.074 | 0.427 | 0.113 (-0.081,0.307) | 0.099 | 0.255 | -0.145 (-0.578,0.288) | 0.221 | 0.512 |
| Fasting insulin | 0.014 (-0.131,0.159) | 0.074 | 0.852 | -0.191 (-0.403,0.021) | 0.108 | 0.078 | -0.111 (-0.491,0.269) | 0.194 | 0.568 |
| Fasting glucose | -0.014 (-0.122,0.094) | 0.055 | 0.797 | -0.019 (-0.164,0.126) | 0.074 | 0.796 | 0.039 (-0.271,0.349) | 0.158 | 0.807 |
| 2hour glucose | -0.078 (-0.305,0.149) | 0.116 | 0.500 | -0.35 (-0.658,-0.042) | 0.157 | 0.026 | -0.043 (-0.696,0.61) | 0.333 | 0.897 |

*****r, genetic correlation of these traits with circulating 25(OH)D or 25(OH)D_3_; 25(OH)D, 25-hydroxyvitamin D; se, standard error of genetic correlation; HOMA-IR, homeostatic model assessment of insulin resistance; HOMA-B, homeostatic model assessment of beta cell function; HbA1c, glycated haemoglobin. PMID refers to the reference ID in the PubMed for the genetic data sources of the traits. *p*<0.007 was considered significant after correction for multiple testing for each 25(OH)D variable.
