# Supplementary material for: The association between circulating 25-hydroxyvitamin D metabolites and type 2 diabetes in European populations: A meta-analysis and Mendelian randomisation analysis
Source: PLoS Med. 2020 Oct 16;17(10):e1003394. doi: 10.1371/journal.pmed.1003394 (PMC7567390; doi:10.1371/journal.pmed.1003394)
Supplement: S4 Table — (DOCX) [file pmed.1003394.s019.docx]

**S4 Table. Observational estimates of 25-hydroxyvitamin D metabolites with incident type 2 diabetes in the EPIC-InterAct study**

|  | Model | n | HR | 95%CIs | | *p value* | Covariates |
| --- | --- | --- | --- | --- | --- | --- | --- |
| Total 25(OH)D | 1 | 20168 | 0.81 | 0.77 | 0.86 | 4.3×10^-15^ | Other covariates* + BMI |
|  | 2 | 16365 | 0.81 | 0.76 | 0.86 | 1.7×10^-11^ | Model 1 + BMI-square + unweighted BMI-GRS |
|  | 3 | 16365 | 0.81 | 0.76 | 0.86 | 9.0×10^-12^ | Model 1 + BMI-square + weighted BMI-GRS |
|  | 4 | 16288 | 0.83 | 0.78 | 0.88 | 3.24×10^-10^ | Model 1 + BMI-square + unweighted BMI-GRS + waist/hip ratio |
| 25(OH)D_3_ | 1 | 20168 | 0.81 | 0.77 | 0.86 | 4.6×10^-15^ | Other covariates* + BMI |
|  | 2 | 16365 | 0.81 | 0.76 | 0.86 | 2.8×10^-11^ | Model 1 + BMI-square + unweighted BMI-GRS |
|  | 3 | 16365 | 0.81 | 0.76 | 0.86 | 1.5×10^-11^ | Model 1 + BMI-square + weighted BMI-GRS |
|  | 4 | 16288 | 0.82 | 0.77 | 0.87 | 1.85×10^-10^ | Model 1 + BMI-square + unweighted BMI-GRS + waist/hip ratio |
| 3-epi-25(OH)D_3_ (binary, above vs below LLQ) | 1 | 20168 | 1.12 | 1.03 | 1.22 | 5.8×10^-03^ | Other covariates* + BMI |
|  | 2 | 16365 | 1.18 | 1.08 | 1.30 | 3.7×10^-04^ | Model 1 + BMI-square + unweighted BMI-GRS |
|  | 3 | 16365 | 1.18 | 1.08 | 1.30 | 3.9×10^-04^ | Model 1 + BMI-square + weighted BMI-GRS |
|  | 4 | 16288 | 1.16 | 1.05 | 1.27 | 2.5×10^-03^ | Model 1 + BMI-square + unweighted BMI-GRS + waist/hip ratio |

*In the EPIC-InterAct study, we used Prentice-weighted Cox regression to estimate the country-specific hazard ratio and 95% CIs for associations per 1-SD difference (calculated from the subcohort) (for C3-epi-25(OH)D_3_: above vs below the LLQ) of plasma total 25(OH)D or metabolites with incident T2D. Other covariates include age as the underlying time-scale, sex, centre, seasonality, physical activity, smoking status, education, alcohol intake, total energy intake, Mediterranean diet score, circulating lipid marker (high-density lipoprotein and low-density lipoprotein cholesterol) and mutual adjustment for other 25(OH)D metabolites. For total 25(OH)D, results from the EPIC-InterAct study in model 1 were pooled with previous published meta-analysis using meta-analysis, which was used in the present study as the observational estimate. For 25(OH)D_3_ and 3-epi-25(OH)D_3_, results from the EPIC-InterAct study in model 1 was directly used as the observational estimates. The BMI-GRS was generated from 97 BMI-related genetic variants by summing up the number of risk alleles; weighted BMI-GRS was created by summing up the number of risk allele and adding the beta coefficient from the GWAS (reference 27 in the text) as the weight for each allele. 25(OH)D, 25-hydroxyvitamin D; HR, hazard ration; CIs, confidence intervals; SD, standard deviation; LLQ, low limit of quantification; GWAS, genome-wide association study; BMI, body mass index; GRS, genetic risk score
