# Supplementary material for: The association between circulating 25-hydroxyvitamin D metabolites and type 2 diabetes in European populations: A meta-analysis and Mendelian randomisation analysis
Source: PLoS Med. 2020 Oct 16;17(10):e1003394. doi: 10.1371/journal.pmed.1003394 (PMC7567390; doi:10.1371/journal.pmed.1003394)
Supplement: S5 Table — (DOCX) [file pmed.1003394.s020.docx]

**S5 Table. Mendelian randomisation analysis of 25-hydroxyvitamin D metabolites with glycaemic traits**

| Glycaemic traits | Selected single nucleotide polymorphisms | Effect size (95%CIs) | *p* value |
| --- | --- | --- | --- |
| **Total 25(OH)D** |  |  |  |
| log (HOMA-IR) | Total 10 SNPs | -0.016 (-0.085, 0.053) | 0.65 |
|  | Four SNPs at previous known genes | -0.012 (-0.08, 0.056) | 0.73 |
|  | Vitamin D synthesis SNPs | 0.039 (-0.081, 0.159) | 0.52 |
|  | Vitamin D metabolism SNPs | -0.037 (-0.12, 0.046) | 0.39 |
|  | Six SNPs at six recently identified genes | -0.037 (-0.197, 0.124) | 0.65 |
| log (HOMA-B) | Total 10 SNPs | -0.025 (-0.077, 0.027) | 0.35 |
|  | Four SNPs at previous known genes | -0.022 (-0.08, 0.035) | 0.44 |
|  | Vitamin D synthesis SNPs | 0.025 (-0.078, 0.129) | 0.63 |
|  | Vitamin D metabolism SNPs | -0.043 (-0.112, 0.025) | 0.21 |
|  | Six SNPs at six recently identified genes | -0.038 (-0.17, 0.094) | 0.57 |
| HbA1C (%) | Total 10 SNPs | 0.038 (0.008, 0.068) | 0.01 |
|  | Four SNPs at previous known genes | 0.037 (0.005, 0.07) | 0.03 |
|  | Vitamin D synthesis SNPs | 0.033 (-0.034, 0.10) | 0.33 |
|  | Vitamin D metabolism SNPs | 0.039 (0.001, 0.076) | 0.04 |
|  | Six SNPs at six recently identified genes | 0.04 (-0.031, 0.111) | 0.27 |
| log (fasting insulin) | Total 10 SNPs | -0.001 (-0.065, 0.064) | 0.98 |
|  | Four SNPs at previous known genes | -0.004 (-0.069, 0.061) | 0.9 |
|  | Vitamin D synthesis SNPs | 0.042 (-0.07, 0.154) | 0.46 |
|  | Vitamin D metabolism SNPs | -0.028 (-0.107, 0.052) | 0.50 |
|  | Six SNPs at six recently identified genes | 0.019 (-0.136, 0.174) | 0.81 |
| Fasting glucose (mmol/L) | Total 10 SNPs | 0.025 (-0.032, 0.082) | 0.39 |
|  | Four SNPs at previous known genes | 0.032 (-0.029, 0.094) | 0.30 |
|  | Vitamin D synthesis SNPs | 0.078 (-0.033, 0.189) | 0.17 |
|  | Vitamin D metabolism SNPs | 0.012 (-0.062, 0.086) | 0.75 |
|  | Six SNPs at six recently identified genes | -0.02 (-0.168, 0.128) | 0.79 |
| 2 hour glucose (mmol/L) | Total 10 SNPs | 0.064 (-0.238, 0.366) | 0.68 |
|  | Four SNPs at previous known genes | -0.017 (-0.267, 0.232) | 0.89 |
|  | Vitamin D synthesis SNPs | 0.154 (-0.178, 0.485) | 0.36 |
|  | Vitamin D metabolism SNPs | -0.239 (-0.617, 0.139) | 0.21 |
|  | Six SNPs at six recently identified genes | 0.787 (0.045, 1.529) | 0.04 |
| **25(OH)D_3_** |  |  |  |
| log (HOMA-IR) | Total seven SNPs | -0.004 (-0.033, 0.026) | 0.81 |
|  | Four SNPs at previous known genes | -0.005 (-0.034, 0.024) | 0.75 |
|  | Vitamin D synthesis SNPs | 0.03 (-0.031, 0.092) | 0.33 |
|  | Vitamin D metabolism SNPs | -0.015 (-0.048, 0.018) | 0.38 |
|  | Three SNPs at three recently identified genes | 0.01 (-0.089, 0.11) | 0.84 |
| log (HOMA-B) | Total seven SNPs | -0.009 (-0.032, 0.014) | 0.45 |
|  | Four SNPs at previous known genes | -0.01 (-0.034, 0.014) | 0.43 |
|  | Vitamin D synthesis SNPs | 0.017 (-0.034, 0.067) | 0.52 |
|  | Vitamin D metabolism SNPs | -0.017 (-0.045, 0.01) | 0.21 |
|  | Three SNPs at three recently identified genes | 0.001 (-0.082, 0.083) | 0.99 |
| HbA1C (%) | Total seven SNPs | 0.015 (0.002, 0.028) | 0.02 |
|  | Four SNPs at previous known genes | 0.015 (0.002, 0.029) | 0.03 |
|  | Vitamin D synthesis SNPs | 0.015 (-0.016, 0.046) | 0.34 |
|  | Vitamin D metabolism SNPs | 0.015 (0.001, 0.03) | 0.04 |
|  | Three SNPs at three recently identified genes | 0.013 (-0.032, 0.059) | 0.56 |
| log (fasting insulin) | Total seven SNPs | 0 (-0.028, 0.028) | 0.97 |
|  | Four SNPs at previous known genes | -0.001 (-0.029, 0.027) | 0.93 |
|  | Vitamin D synthesis SNPs | 0.033 (-0.026, 0.093) | 0.27 |
|  | Vitamin D metabolism SNPs | -0.011 (-0.043, 0.02) | 0.49 |
|  | Three SNPs at three recently identified genes | 0.021 (-0.075, 0.116) | 0.67 |
| Fasting glucose (mmol/L) | Total seven SNPs | 0.013 (-0.012, 0.038) | 0.30 |
|  | Four SNPs at previous known genes | 0.013 (-0.013, 0.04) | 0.32 |
|  | Vitamin D synthesis SNPs | 0.045 (-0.012, 0.102) | 0.12 |
|  | Vitamin D metabolism SNPs | 0.005 (-0.025, 0.034) | 0.75 |
|  | Three SNPs at three recently identified genes | 0.013 (-0.078, 0.104) | 0.79 |
| 2 hour glucose (mmol/L) | Total seven SNPs | -0.009 (-0.219, 0.201) | 0.93 |
|  | Four SNPs at previous known genes | -0.032 (-0.159, 0.095) | 0.62 |
|  | Vitamin D synthesis SNPs | 0.123 (-0.113, 0.36) | 0.31 |
|  | Vitamin D metabolism SNPs | -0.096 (-0.246, 0.055) | 0.21 |
|  | Three SNPs at three recently identified genes | 0.297 (-0.161, 0.754) | 0.20 |
| **C3-epi-25(OH)D_3_ (binary)** |  |  |  |
| log (HOMA-IR) | Total three SNPs | 0.004 (-0.045, 0.053) | 0.87 |
|  | *SDR9C7* SNP | 0.042 (-0.027, 0.11) | 0.23 |
|  | The other SNPs | -0.007 (-0.045, 0.03) | 0.71 |
| log (HOMA-B) | Total three SNPs | -0.007 (-0.04, 0.026) | 0.67 |
|  | *SDR9C7* SNP | 0.008 (-0.05, 0.066) | 0.78 |
|  | The other SNPs | -0.012 (-0.043, 0.019) | 0.46 |
| HbA1C (%) | Total three SNPs | 0.018 (0.003, 0.034) | 0.02 |
|  | *SDR9C7* SNP | 0.018 (-0.016, 0.051) | 0.30 |
|  | The other SNPs | 0.019 (0.001, 0.036) | 0.04 |
| log (fasting insulin) | Total three SNPs | 0.004 (-0.036, 0.044) | 0.85 |
|  | *SDR9C7* SNP | 0.019 (-0.046, 0.084) | 0.57 |
|  | The other SNPs | -0.001 (-0.037, 0.036) | 0.97 |
| Fasting glucose (mmol/L) | Total three SNPs | 0.027 (-0.009, 0.063) | 0.14 |
|  | *SDR9C7* SNP | 0.066 (0.004, 0.129) | 0.04 |
|  | The other SNPs | 0.015 (-0.019, 0.049) | 0.38 |
| 2 hour glucose (mmol/L) | Total three SNPs | -0.024 (-0.199, 0.151) | 0.79 |
|  | *SDR9C7* SNP | 0.091 (-0.241, 0.422) | 0.59 |
|  | The other SNPs | -0.055 (-0.228, 0.118) | 0.53 |

Mendelian randomization (MR) estimate represents the association between a genetically predicted increase of 1-standard deviation in 25-hydroxyvitamin D metabolites (except for the binary C3-epi-25(OH)D_3_ variable: above vs below lower limit of quantification) and glycaemic traits. p-value<0.008 was considered significant for each vitamin D variable after correction for multiple testing.

For total 25(OH)D, four prior known genes are *GC* (rs3755967), *CYP2R1* (rs116970203), *NADSYN1/DHCR7* (rs12785878), and *CYP24A1* (rs17216707). Two genes in the 25(OH)D synthesis pathway are *CYP2R1* (rs116970203) and *NADSYN1/DHCR7* (rs12785878). Two genes related to the 25(OH)D metabolism are *GC* (rs3755967) and *CYP24A1* (rs17216707). Six recent identified genes are *PADI1*, *CRCT1*, *UGT1A5*, *AMDHD1*, *SEC23A* and *SULT2A1 identified in the present GWAS-meta-analysis*.

For 25(OH)D_3_, four prior known genes are *GC* (rs4588), *CYP2R1* (rs116970203), *NADSYN1/DHCR7* (rs28364617), and *CYP24A1* (rs17216707). Two genes in the 25(OH)D synthesis pathway are *CYP2R1* (rs116970203) and *NADSYN1/DHCR7* (rs28364617). Two genes related to the 25(OH)D metabolism are *GC* (rs4588) and *CYP24A1* (rs17216707). Three recent identified genes are *SHQ1*, *AMDHD1* and *SULT2A1* identified in the present GWAS-meta-analysis.

For C3-epi-25(OH)D_3_, we did sensitivity analysis stratified by *SDR9C7* variant and the other variants (related to total 25(OH)D), as *SDR9C7* is a unique locus associated with C3-epi-25(OH)D_3_. The *SDR9C7* SNP was rs11172066.

SNP, single nucleotide polymorphism; 25(OH)D, 25-hydroxyvitamin D; HOMA-IR, homeostatic model assessment of insulin resistance; HOMA-B, homeostatic model assessment of beta cell function; HbA1c, glycated haemoglobin. CIs, confidence intervals. GWAS, genome-wide association study.
