# Supplementary material for: The association between circulating 25-hydroxyvitamin D metabolites and type 2 diabetes in European populations: A meta-analysis and Mendelian randomisation analysis
Source: PLoS Med. 2020 Oct 16;17(10):e1003394. doi: 10.1371/journal.pmed.1003394 (PMC7567390; doi:10.1371/journal.pmed.1003394)
Supplement: S1 Text — (DOCX) [file pmed.1003394.s021.docx]

**S1 Text. study protocol**

***15 Aug 2016***

**Study protocol for the analysis of nutritional biomarkers (including vitamin D biomarkers) and type 2 diabetes**

Circulating 25-hydroxyvitamin D, vitamin C and carotenoids: observational associations with and causal effects on type 2 diabetes-EPIC-InterAct Study

**Research question**

Blood 25-hydroxyvitamin D [25(OH)D] reflects vitamin D status. Observational studies have shown its inverse association with T2D risk, but the association seems unlikely to be causal based on our Mendelian randomisation analysis (Ye et al, Lancet Diabetes Endocrinol 2015). However, the analysis was based on four SNPs previously identified to be related with 25(OH)D (Wang TJ et al, Lancet 2010) that together explained <4% of the variation in 25(OH)D concentrations. This warrants further discovery efforts. Moreover, environmental determinants of circulating 25(OH)D have been poorly explored across countries of Europe.
Blood vitamin C and carotenoids reflect dietary fruit and vegetable intake, and are inversely associated with T2D risk (Harding et al, Arch Int Med 2008; Cooper et al, EJCN 2015). However, large-scale prospective evidence is limited. Further, causality of the association of exposure to vitamin C and carotenoids with T2D risk has not been investigated so far.
The ‘Cambridge Initiative’ funded the measurements of circulating 25(OH)D, vitamin C and carotenoids in InterAct, providing a unique opportunity to investigate their associations with incident T2D.

**Objectives**

1. To describe the distribution of each of plasma 25(OH)D, vitamin C and carotenoids across European countries, and examine their cross-sectional associations with various sociodemographic and lifestyle, including dietary variables.
2. To estimate the association of plasma 25(OH)D, vitamin C and carotenoids with T2D and to estimate the causal effects on T2D by using Mendelian randomisation (MR).

**Main exposures**

Plasma 25(OH)D [total, D2, D3 and isomers], plasma vitamin C and carotenoids (total, alpha-carotene, beta-carotene, beta-cryptoxanthin, lycopene, lutein, zeaxanthin). SNPs associated with plasma 25(OH)D, vitamin C and carotenoids (these SNPs will be identified through the GWAS of InterAct and other available studies).

**Main potential confounding factors**

Country, centre, age, sex, BMI, smoking, alcohol intake, education, physical activity, medical history (cancer, CHD, stroke), month of blood collection, total cholesterol, HDL-cholesterol, LDL-cholesterol, lipid-lowering medication, dietary factors (vegetable and fruit intake, fish intake, egg intake, milk and dairy product intake, calcium intake, energy intake, fat, saturated fat).

**Other specific analytical issues**

For the MR analysis
(1) SNP/biomarker level associations:
a. 25(OH)D-related SNPs will be identified by conducting GWAS in InterAct, as well as other studies where 25(OH)D and GWAS are available (e.g. EPIC-Norfolk and Ely study, excluding any duplicate samples).
b. Vitamin C-related SNPs will be identified by conducting GWAS in InterAct, as well as other studies where plasma vitamin C and GWAS is available (e.g. EPIC-Norfolk study; Fenland study, excluding duplicated samples). For carotenoids, SNPs will be identified by GWAS in InterAct and EPIC-Norfolk.
(2) SNP/T2D association: Results from InterAct will be used, together with data from other sources (including DIAGRAM, Norfolk Diabetes Study, ADDITION-Ely Study and Cambridgeshire Case-control Study).

**Analysis Plan**

**(1) Cross-sectional distribution and correlates of 25(OH)D, vitamin C and carotenoids at baseline**
Skewed variables will be log-transformed. Linear regression coefficients between 25(OH)D and latitude will be calculated, adjusted for age, BMI and physical activity. Previous studies have reported an unexpected positive correlation that higher latitude (with less sunlight exposure) was associated with higher level of 25(OH)D [1, 2]. To interpret the potential positive 25(OH)D-latitude association, distribution of other potential determinants of 25(OH)D (dietary vitamin D intake, BMI and physical activity) by study centre will also be analysed. Centre-specific linear regression coefficients describing the potential determinants of 25(OH)D will be calculated. The same analyses will be performed for potential food sources (fish, egg, milk, and other dairy products).
Country-specific linear regression coefficients of plasma vitamin C and carotenoids with potential determinants will be estimated, adjusting for study centre, age, sex, smoking and energy intake, and then pooled via random-effects meta-analysis. Distribution of plasma vitamin C and carotenoids across different countries will be displayed and potential determinants of the concentration of each biomarker will be explored. Association of 25(OH)D, vitamin C and carotenoids with some selected biomarkers including albumin, calcium, creatinine, glucose, HbA1c, hsCRP, triglycerides, blood pressure and pulse variables, will be examined.
**(2) Analysis of associations of each biomarker with incident T2D**
To investigate the association of plasma 25(OH)D, vitamin C and carotenoids with T2D, Prentice-weighted Cox regression will be used, with age as the underlying time-scale and incident T2D as the outcome. Analysis will be performed per country with each biomarker modelled both in quintiles and as a continuous variable. The estimated associations will be pooled across countries via random-effects meta-analysis. The following covariates will be included in a model sequentially:
a) Demographic variables: age, sex, study centre, month of blood collection
b) Lifestyle and social factors and medical history: smoking status, education level, physical activity, family history of diabetes, prevalent cancer, prevalent CHD, prevalent stroke
c) Dietary factors: energy intake, alcohol intake and a diet quality score (eg, the Mediterranean diet score)
d) BMI will be treated as a confounder and will be tested for its influence on the measure of association.

**(3) Genome-wide association study (GWAS)**
GWAS will be performed within InterAct participants (discovery cohort) to identify genetic variants associated with plasma 25(OH)D, vitamin C and carotenoids. Then, analyses for replication will be performed by using data from EPIC-Norfolk and Ely study for 25(OH)D; EPIC-Norfolk and Fenland Study for plasma vitamin C; and EPIC-Norfolk study for carotenoids (separate permissions will be sought for these data). Final results will be meta-analysed from different studies for each biomarker with fixed-effect meta-analysis model.
**(4) Mendelian randomisation (MR) analysis**
MR will be performed by combining two measures of association, as previously performed [3]. We will estimate causal effect of each biomarker on T2D (MR estimate) by dividing the instrumental variable- biomarker estimate, and the instrumental variable-T2D association: (1) SNP/biomarker level association: SNPs related to 25(OH)D, vitamin C and carotenoids will be identified by conducting GWAS in InterAct and replicated in other studies mentioned above. (2) For the associations between SNPs and T2D risk, results from InterAct will be used, together with data from other sources, including DIAGRAM, Norfolk Diabetes Study, ADDITION-Ely Study and Cambridgeshire Case-control Study. Assumptions of MR analysis will be assessed by analysing associations of selected genetic variables with demographics, lifestyle, and other factors related to incident T2D.

Addition on 8 July 2020: Different MR methods were used to estimate the overall association. We kept inverse-variance weighted (IVW) MR as the primary approach. The other methods were considered as secondary approaches and conducted post hoc: MR-Egger, weighted median, MR-PRESSO [4], MR-RAPS[5], MRMix[6] and multivariable MR method[7]. We recognise IVW, MR-Egger and weighted median as the most used methods in the literature. MR-PRESSO aims to estimate a causal effect robust against horizontal pleiotropy, suited when horizontal pleiotropy occurs in <50% of instruments; MR-RAPS extends the IVW method in a way similar to robust regression by weighting variants in a function of the causal effect of interest; MRMix accounts for the probabilities of genetic instruments’ fit to different causal mechanisms and is believed to provide nearly unbiased or/and less biased estimates of causal effects compared to other methods. Multivariable MR uses multiple genetic variants associated with several measured risk factors to simultaneously estimate the causal effect independent of each of the risk factors on the outcome.

References:
1. van Schoor NM and Lips P, Worldwide vitamin D status. Best Pract Res Clin Endocrinol Metab, 2011. 25(4): p. 671-80.
2. Mithal A, Wahl DA, Bonjour JP, et al., Global vitamin D status and determinants of hypovitaminosis D. Osteoporos Int, 2009. 20(11): p. 1807-20.
3. Burgess S, Butterworth A and Thompson SG, Mendelian Randomization Analysis With Multiple Genetic Variants Using Summarized Data. Genetic Epidemiology, 2013. 37(7): p. 658-65.

4.Verbanck M, Chen CY, Neale B, Do R. Detection of widespread horizontal pleiotropy in

causal relationships inferred from Mendelian randomization between complex traits and

diseases. Nat Genet. 2018; doi:10.1038/s41588-018-0099-7

5.Zhao Q, Wang J, Hemani G, Bowden J, Small DS. Statistical inference in two-sample

summary-data Mendelian randomization using robust adjusted profile score.

arXiv:180109652. 2018; Available: http://arxiv.org/abs/1801.09652

6.Qi G, Chatterjee N. Mendelian randomization analysis using mixture models for robust and

efficient estimation of causal effects. Nat Commun. 2019; doi:10.1038/s41467-019-09432-2

7.Burgess S, Thompson SG. Multivariable Mendelian randomization: The use of pleiotropic

genetic variants to estimate causal effects. Am J Epidemiol. 2015; doi:10.1093/aje/kwu283

**Person responsible for data analysis**

Ju-Sheng Zheng

***25 May 2017***

**Analysis plan: GWAS for plasma nutritional biomarkers**

**EPIC-Norfolk (plasma vitamin D)**

Biomarker list: **plasma 25(OH)D3, 25(OH)D2, 3-epi-25(OH)D3, 3-epi-25(OH)D3%, total 25(OH)D (25(OH)D3+25(OH)D2)**

Phenotype data preparation (n~11438 for vitamin D)

1. Exclude participants, which were also included in the InterAct study and EPIC-CVD (to avoid overlap of participants with InterAct study and EPIC-CVD)
2. Winsorise variables at top and bottom 0.1%
3. Ln-transform for 25(OH)D metabolites
4. For samples with GWAS data, calculate standardised residuals for the transformed variables
   1. adjusted for age, sex, BMI, season of blood collection

The name for the 25(OH)D metabolites are:

‘ln_vitd’ ‘ln_vitd3’ ‘ln_vitd2’ ‘ln_vitd3_epi’ ‘ln_vitd3_epi_p’

The name for other confounders are ‘age’, ‘sex’ (1- male; 2=female), ‘bmi’, and ‘season’ (season of blood collection)

**InterAct (plasma vitamin C, 25(OH)D and carotenoids)**

Biomarker list: **plasma vitamin C, beta-carotene, alpha-carotene, lutein, beta-cryptoxanthin, lycopene, zeaxanthin, total carotenoids (sum of the six carotenoids), 25(OH)D3, 25(OH)D2, 3-epi-25(OH)D3, 3-epi-25(OH)D3%, total 25(OH)D (25(OH)D3+25(OH)D2)**

Phenotype data preparation

1. For biomarkers below the lower limit of detection (LLD), create a random value between 0 and the LLD cut-off point. (Note: Missing data of 25(OH)D2 and 3-epi-25(OH)D3 will not be imputed as more than 50% of the data for the two biomarkers were below LOD)
2. Winsorise variables at top and bottom 0.1%
3. Ln-transform for all individual and total carotenoids and all 25(OH)D
4. For samples with GWAS data, calculate standardised residuals for the transformed/or untransformed variables, stratified by case-subcohort status (all countries’ samples together)
   1. adjusted for age, sex, i.centre
   2. adjusted for age, sex, i.centre, BMI, season of blood collection (for 25(OH)D only), smoking (never, former, current, for plasma vitamin C and carotenoids)

For InterAct datasets, the name for plasma vitamin C is ‘vitc’, for carotenoids (ln-transformed) are ‘ln_lutein’ ‘ln_zeax’ ‘ln_bcrypt’ ‘ln_acarot’ ‘ln_bcarot’ ‘ln_lycop’ ‘ln_tot_carot’, for 25(OH)D (ln-transformed) are ‘ln_vitd3’ ‘ln_vitd2’ ‘ln_vitd3_epi’ ‘ln_vitd3_epi_p’ ‘ln_vitd’.

The name for other confounders are ‘age’, ‘sex’ (1- male; 2=female), ‘centre’, ‘bmi’, ‘smoke’(1=never, 2-former, 3=smoker) and ‘season’ (season of blood collection)

The name for the variable to separate subcohort and non-subcohort is ‘outcome’ (0=non-subcohort, 1=subcohort)

**GWAS analyses**

Outcome variables: all three categories of nutritional biomarkers (vitamin C, 25(OH)D and carotenoids).

Run linear regression (additive) models in **subcohort and non-subcohort** separately, and 660w and and Core-exome chips separately, using HRC imputed data, adjusting for 10PCs as the only covariates (we already accounted for age, sex, BMI and centre).

**Post-GWAS analysis QC:**

Keep markers with: MAC >= 10, Imputation_info >= 0.4 and SE<=10

Meta-analyse two chips, and subcohort and non-subcohort

Genome control adjustment pre meta-analysis, in metal, Genomecontrol ON

Clump results where p < 0.05

Output: Generate a QQ plot (overall, and excluding known loci) and table of SNPs where p < 10^-5.^  Also include heterogeneity estimates.

**EPIC-CVD, modified based on InterAct analysis plan**

Biomarker list: **plasma vitamin C, beta-carotene, alpha-carotene, lutein, beta-cryptoxanthin, lycopene, zeaxanthin, total carotenoids (sum of the six carotenoids), 25(OH)D3, 25(OH)D2, 3-epi-25(OH)D3, 3-epi-25(OH)D3%(ratio of 3-epi-25(OH)D3 to 25(OH)D3)**

Phenotype data preparation

1. Winsorise variables at top and bottom 0.1% (calculated based on the subcohort)
2. Excluding all the participants overlapped with InterAct
3. For vitamin C and carotenoids GWAS, exclude all the participants from EPIC-Norfolk.
4. Ln-transform for all individual and total carotenoids and all 25(OH)D metabolites
5. For samples with GWAS data, calculate standardised residuals for the transformed/or untranformed variables, stratified by case-subcohort status (all countries’ samples together)
   1. For vitamin C and carotenoids: adjusted for age, sex, study centre
   2. For 25(OH)D: adjusted for age, sex, study centre, BMI and season of blood collection (Winter [Dec, Jan, Feb], Spring [Mar, Apr, May], Summer [June, July, Aug], Autumn [Sept, Oct, Nov])

**GWAS analyses**

Outcome variables: all three categories of nutritional biomarkers (vitamin C, 25(OH)D metabolites and carotenoids).

Run linear regression (additive) models in **subcohort and non-subcohort** separately, and chip separately (if there are multiple chips), using HRC imputed data, adjusting for 10 PCs as the only covariates (we already accounted for age, sex, centre, season (vitamin D) and BMI (vitamin D)).

The GWAS results will be combined via fixed-effect inverse-variance weighted meta-analysis.

***30 Oct 2017***

**Additional analysis plan: GWAS for plasma vitamin D metabolites in three studies (EPIC-Norfolk, EPIC-InterAct and EPIC-CVD)**

***[Note – a previous analysis plan was agreed in May 2017, and this additional plan supplements the original analysis plan, taking account of the high missingness on the vitamin D2 and the c3-epimer metabolites]***

**EPIC-Norfolk Study**

Biomarker list: **25(OH)D2, 3-epi-25(OH)D3**

Phenotype data preparation (n~11438 for vitamin D from 2^nd^ health check phase)

1. Exclude participants who were also included in the InterAct and EPIC-CVD studies (to avoid overlap of participants across these studies)
2. Winsorise variables at top and bottom 0.1%
3. Create binary 25(OH)D variable (above versus below lower limit of quantification)

The name for the 25(OH)D metabolites are:

‘bi_vitd2’(1= above LLD, 0= below LLD)

‘bi_vitd3_epi’ (1=above LLD, 0= below LLD)

The variable names for potential confounders are ‘age’, ‘sex’ (1- male; 2=female), ‘bmi’, and ‘season’ (season of blood collection)

**GWAS analyses**

Run logistic regression (additive) models using HRC imputed data, adjusting for 10PCs, age, sex, BMI and season of blood collection.

**EPIC-InterAct Study**

Biomarker list: **25(OH)D2, 3-epi-25(OH)D3**

Phenotype data preparation

1. Exclude those included in EPIC-Norfolk study (to avoid overlap)
2. For biomarkers below the lower limit of detection (LLD), create a random value between 0 and the LLD cut-off point. (Note: Missing data of 25(OH)D2 and 3-epi-25(OH)D3 will not be imputed as more than 50% of the data for the two biomarkers were below LOD)
3. Winsorise variables at top and bottom 0.1%
4. Create binary 25(OH)D variable (above versus below lower limit of quantification)

For InterAct datasets, the name for 25(OH)D are

‘bi_vitd2’ (1= above LLD, 0= below LLD)

‘bi_vitd3_epi’(1= above LLD, 0= below LLD)

The variable names for potential confounders are ‘age’, ‘sex’ (1- male; 2=female), ‘centre’, ‘bmi’, and ‘season’ (season of blood collection)

The variable name to separate subcohort and non-subcohort is ‘outcome’ (0=non-subcohort, 1=subcohort)

**GWAS analyses**

Run logistic regression (additive) models in **subcohort and non-subcohort** separately, and 660w and Core-exome chips separately, using HRC imputed data, adjusting for 10PCs, age, sex, BMI, study centre and season of blood collection.

**EPIC-CVD Study**

Biomarker list: **25(OH)D2, 3-epi-25(OH)D3**

Phenotype data preparation

1. Winsorise variables at top and bottom 0.1% (calculated based on the subcohort).
2. Exclude all the participants overlapping with InterAct or EPIC-Norfolk [but note that EPIC-CVD will include subcohort participants from Norway and Greece that were not part of the original InterAct subcohort].
3. Create binary 25(OH)D variable (above versus below lower limit of quantification)

**GWAS analyses**

Run logistic regression (additive) models in **subcohort and non-subcohort** separately, using HRC imputed data, adjusting for 10PCs, age, sex, study centre, BMI and season of blood collection.

The GWAS results will be combined via fixed-effect inverse-variance weighted meta-analysis.

***14 May 2019***

**Analysis plan for UK biobank**

**UK biobank for total 25(OH)D**

Phenotype data preparation

1. Winsorise variables at top and bottom 0.1%
2. Ln-transform for total 25(OH)D
3. For samples with GWAS data, calculate standardised residuals for the transformed variables, adjusted for age, sex, BMI, season of blood collection

The name for the 25(OH)D is:

‘ln_vitd’

The name for other confounders are ‘age’, ‘sex’ (1- male; 2=female), ‘bmi’, and ‘season’ (season of blood collection)

**GWAS analyses**

Outcome variables: total 25(OH)D

Run linear regression (additive) models in using HRC imputed data, adjusting for 10 PCs as the only covariates (we already accounted for age, sex, centre, season and BMI).

**Mendelian randomisation (MR) analysis**

The MR analysis plan was documented in the original EPIC-InterAct analysis plan. We used an established pipeline within the MRC Epidemiology Unit to run the MR analysis using three most commonly used MR methods: IVW, MR-Egger and weighted median method. We planned to use the available summary statistics of type 2 diabetes GWAS meta-analysis hold in the MRC Epidemiology unit.

The analysis was planned and performed in Dec 2017 after the GWAS analyses, and updated analyses on this was performed in Jan 2019 to increase the sample size of the genetic variant-T2D associations.

**Observational association of vitamin D metabolites and type 2 diabetes incidence.**

The analysis was documented in the original EPIC-InterAct analysis plan. The observational association of vitamin D metabolites and type 2 diabetes in EPIC-InterAct was analysed before 2018 and published online in Nov 2018 (Zheng et al, J Clin Endocrinol Metab 2019, 104(4): 1293-1303.).

We took the effect estimate from the above published EPIC-InterAct paper and combined it with other published results using random-effects meta-analysis. The analyses were planned and performed after getting the results of the above 2 stages (GWAS and MR analysis) in Jan 2018.
