# Supplementary material for: The association between circulating 25-hydroxyvitamin D metabolites and type 2 diabetes in European populations: A meta-analysis and Mendelian randomisation analysis
Source: PLoS Med. 2020 Oct 16;17(10):e1003394. doi: 10.1371/journal.pmed.1003394 (PMC7567390; doi:10.1371/journal.pmed.1003394)
Supplement: S2 Text — (DOCX) [file pmed.1003394.s022.docx]

**S2 Text. Description of the participating studies in the present genome-wide association study for vitamin D metabolites**

**EPIC-InterAct study**

EPIC-InterAct is a case-cohort study nested within the European Prospective Investigation into Cancer and Nutrition study(1), including 12,403 incident type 2 diabetes cases verified from 340,234 participants across eight European countries (France, Italy, Spain, UK, Netherlands, Germany, Sweden and Denmark), and 16,154 subcohort members (including 778 verified incident type 2 diabetes cases as a feature of the case-cohort design) randomly selected from these participants. Ascertainment of incident type 2 diabetes cases up until Dec 31, 2007, was conducted through a review of multiple sources of evidence, including self-report, linkage to primary care registers, secondary care registers, medication use (drug registers), hospital admissions and mortality data. There were no type 2 diabetes cases ascertained solely by self-report and we sought further evidence for all cases with information on incident type 2 diabetes from fewer than two independent sources at minimum. Type 2 diabetes cases in Denmark and Sweden were identified through local and national diabetes and pharmaceutical registers and were considered to be verified.

In the EPIC-InterAct study, genome-wide genotyping was performed among 23,019 participants with the Illumina HumanCoreExome array (n=13,725) or Illumina 660W-Quad BeadChip (n=9,294). After exclusion of those without vitamin D measurements, a total of 18,078 participants in the EPIC-InterAct contributed to the GWAS of 25(OH)D and 25(OH)D_3_. The GWAS in EPIC-InterAct was conducted stratified by the subcohort status (subcohort and non-subcohort) and the GWAS arrays used, thus four GWAS were performed within the EPIC-InterAct study.

**EPIC-Norfolk**

Between 1993 and 1997, a total of 25,639 men and women aged 40-79 were recruited into the EPIC-Norfolk study, one of the two UK constituents of the European Prospective Investigation into Cancer and Nutrition study(2). Genome-wide genotyping in EPIC-Norfolk was conducted among 21,044 participants using the Affymetrix UK Biobank Axiom Array. We excluded the EPIC-Norfolk case-cohort study (n=1503) which was part of the EPIC-InterAct study to avoid duplication with the EPIC-InterAct study for the GWAS analysis. After further excluding those without measurement of blood vitamin D, 10,231 participants were included in the GWAS of 25(OH)D and 25(OH)D_3_.

**EPIC-CVD study**

EPIC-CVD study is a large, prospective, case-cohort study nested within the European Prospective Investigation into Cancer and Nutrition study, including participants from ten European countries: France, Italy, Spain, UK, Netherlands, Germany, Sweden, Denmark, Norway and Greece(3). EPIC-CVD contains a random subcohort of 18,249 participants and 24,557 participants who later developed cardiovascular diseases during the follow-up. Genome-wide genotyping in the EPIC-CVD study was conducted using Illumina Human Exome v1.1 SNP array. EPIC-CVD study shared the subcohort with the EPIC-InterAct study and therefore those overlapping individuals were excluded for the vitamin D GWAS, and the GWAS was performed separately in the subcohort (n=887) and non-subcohort (n=11,366) samples, with a total of 12,253 participants.

**Ely study**

The Ely study was established in 1990 as a prospective study among adults free of known diabetes and registered with a single practice serving Ely, with 1,122 adults of European origin included at baseline(4). Genome-wide genotyping was performed by using the Illumina HumanCoreExome array. A total of 690 participants in the Ely study had both genotype data and blood vitamin D measurement and were included in the present GWAS analyses.

**UK Biobank**

The UK Biobank study is a population-based cohort of half a million UK individuals aged 40-69 years recruited between 2006 and 2010 across the UK(5). Among the total 487,409 UK Biobank participants with genotype data, 35,098 non-European-descent participants and 64 withdraw samples were excluded. Furthermore, 41,421 samples with missing values in blood 25(OH)D or covariates were also excluded. Finally, a total of 410,826 samples were included in the GWAS analysis of total 25(OH)D, which was used as a replication dataset after GWAS discover.

**Description of the genome-wide genotyping methods and participating studies in the genome-wide association studies for type 2 diabetes**

In the EPIC-InterAct study, genome-wide genotyping was performed with Illumina HumanCoreExome array or Illumina 660W-Quad BeadChip. In the EPIC-Norfolk study and the UK Biobank study, Affymetrix UK Biobank Axiom Array was used for the genome-wide genotyping, and in the EPIC-CVD study and Ely study, Illumina HumanCore Exome array was used for the genome-wide genotyping. For the above studies (EPIC-InterAct, EPIC-Norfolk, EPIC-CVD and Ely), genotype imputation was performed to the Haplotype Reference Consortium reference panel using IMPUTE v2 software.

**UK Biobank**

The UK Biobank study is a population-based cohort of half a million UK individuals aged 40-69 years recruited between 2006 and 2010 across the UK(5). Both genotype data and prevalent/incident type 2 diabetes information was available among a total 449,333 individuals in the initial UK Biobank dataset (24,758 cases and 424,575 controls). Type 2 diabetes was defined by self-reported physician diagnosis at nurse interview or digital questionnaire, age at diagnosis>36 years, and use of oral anti-diabetic medications.

**DIAbetes Meta-ANalysis of Trans-Ethnic association studies (DIAMANTE) (European)**

DIAMANTE is a consortium which published meta-analysis of genome-wide association studies of type 2 diabetes in individuals of different ethnicities, and the meta-analysis results from DIAMANTE (European ethnicity) including a total of 74,124 T2D cases and 824,006 controls were published and publicly available(6). In the present study, we used the summary statistics from the DIAMANTE European excluding UK Biobank study, thus including 55,005 T2D cases and 400,308 controls.

**EPIC-Norfolk**

We used the T2D GWAS results from EPIC-Norfolk excluding T2D cases already included in the DIAMANTE study, and finally included 1,220 T2D cases and 18,026 controls.

The summary statistics (80,983 T2D cases and 842,909 controls) from the meta-analysis of the GWAS from UK Biobank, DIAMANTE and EPIC-Norfolk was used in the present study to examine the genetic association of vitamin D variants with T2D risk. The EPIC-Norfolk T2D GWAS that contributed to the meta-analysis of DIAMANTE + UKBB + EPIC-Norfolk, was based on HRC + UK10K+1000G imputation, not just HRC imputation.

**References:**

1. InterAct Consortium, Langenberg C, Sharp S, Forouhi NG, Franks PW, Schulze MB, Kerrison N, Ekelund U, Barroso I, Panico S, et al. Design and cohort description of the InterAct Project: an examination of the interaction of genetic and lifestyle factors on the incidence of type 2 diabetes in the EPIC Study. Diabetologia. 2011;54:2272–82.

2. Day N, Oakes S, Luben R, Khaw KT, Bingham S, Welch A, Wareham N. EPIC-Norfolk: study design and characteristics of the cohort. European Prospective Investigation of Cancer. Br J Cancer. 1999;80 Suppl 1:95–103.

3. Danesh J, Saracci R, Berglund G, Feskens E, Overvad K, Panico S, Thompson S, Fournier A, Clavel-Chapelon F, Canonico M, et al. EPIC-Heart: The cardiovascular component of a prospective study of nutritional, lifestyle and biological factors in 520,000 middle-aged participants from 10 European countries. Eur J Epidemiol. 2007;

4. Forouhi NG, Luan J, Hennings S, Wareham NJ. Incidence of Type 2 diabetes in England and its association with baseline impaired fasting glucose: The Ely study 1990-2000. Diabet Med. 2007;

5. Collins R. What makes UK Biobank special? The Lancet. 2012.

6. Mahajan A, Taliun D, Thurner M, Robertson NR, Torres JM, Rayner NW, Payne AJ, Steinthorsdottir V, Scott RA, Grarup N, et al. Fine-mapping type 2 diabetes loci to single-variant resolution using high-density imputation and islet-specific epigenome maps. Nat Genet. Nature Publishing Group; 2018;50:1505–13.
